# *Mycobacteriales* taxonomy using network analysis-aided, context-uniform phylogenomic approach for non-subjective genus demarcation

**DOI:** 10.1128/mbio.02207-23

**Published:** 2023-10-05

**Authors:** Jorge Val-Calvo, José A. Vázquez-Boland

**Affiliations:** 1 Microbial Pathogenesis Laboratory, Infection Medicine, Edinburgh Medical School (Biomedical Sciences), University of Edinburgh, Edinburgh, Scotland, United Kingdom; Washington University in St. Louis, St. Louis, Missouri, USA; Washington University School of Medicine, St. Louis, Missouri, USA

**Keywords:** *Mycobacteriales *phylogenomics, prokaryotic genus definition, genus demarcation, *Rhodococcus*, *Rhodococcus equi*, *Prescottella*, *Rhodococcoides*, *Rhodococcoides fascians*, *Mycobacterium*, *Mycobacteroides*, *Mycobacteriineae*, *Corynebacteriineae*

## Abstract

Genomics allows accurately pinpointing microbial ancestry for taxonomic purposes, yet there is currently no consensus to genus definition based on genome data. Different metrics and criteria are used, with an increasing trend toward genera over-splitting. Here, we report a method for prokaryotic genus assignment that combines normalized tree clustering and network analysis of several genomic relatedness indices. Objectivity is maximized by linear application of the same partitioning thresholds across an entire middle/high taxonomic rank context (order level), with the classical (“pre-genomic”) genera as demarcation reference, ensuring continuity in taxonomy and nomenclature. We tested the method with the *Mycobacteriales*, where recent examples of genus fragmentation divided *Mycobacterium* into five genera, or made *Rhodococcus* paraphyletic by creating the genus *Prescottella* for the sublineage containing the animal and human pathogen, *Rhodococcus equi*. Our approach did not support the mycobacterial five-genus split or the *Prescottella* nested genus, but identified a basal branch in each of the mycobacterial and rhodococcal radiations warranting genus status (*Mycobacteroides*, and novel genus *Rhodococcoides* for the *Rhodococcus fascians* clade, respectively). The median average amino acid identity (AAI) between the demarcated genera was 56% to 59%, consistent with the <65% AAI genus boundary standard. Shifting the demarcation threshold to the *Prescottella*/mycobacterial five-genus level systematically elevated the intrageneric sublineages to genus rank, leading to taxonomic atomization (≈threefold increase in potential *Mycobacteriales* genera). The proposed approach provides a standardizable methodological framework for non-subjective prokaryotic genus delineation.

**IMPORTANCE:**

A robust taxonomy is essential for the organized study of prokaryotes and the effective communication of microbial knowledge. The genus rank is the mainstay of biological classification as it brings together under a common name a group of closely related organisms sharing the same recent ancestry and similar characteristics. Despite the unprecedented resolution afforded by whole-genome sequencing in defining evolutionary relationships, a consensus approach for phylogenomics-based prokaryotic genus delineation remains elusive. Taxonomists use different demarcation criteria, sometimes leading to genus rank over-splitting and the creation of multiple new genera. This work reports a simple, reliable, and standardizable method that seeks to minimize subjectivity in genomics-based demarcation of prokaryotic genera, exemplified through application to the order *Mycobacteriales*. Formal descriptions of proposed taxonomic changes based on our study are included.

## INTRODUCTION

As a classification method, prokaryotic taxonomy (or systematics) and nomenclature are meant to facilitate the organized understanding of microbes “*by providing information unknown to the user but relating to something the name of which is known*” (1). The genus rank embodies the essence of this concept because it reunites under the same name a group of closely related organisms below family and above species level sharing similar biological characteristics and close evolutionary relationships. The genus occupies a central position in the taxonomic hierarchy and critically influences the nomenclature of the higher ranks, from family to phylum (2, 3). Unfortunately, whereas the way to determine a species is reasonably clear and standardized (4 – 11), there are currently no unanimously accepted criteria for objective prokaryotic genus definition based on genomic data (8, 12 – 16). The matter is thus largely left at the discretion of taxonomic interpretation and is a source of ongoing debate.

An illustrative example in the field of pathogenic bacteria is the recent subdivision of the genus *Mycobacterium* based on phylogenomic analyses (17). Four new genera, i.e. *Mycobacteroides*, *Mycolicibacter, Mycolicibacillus*, and *Mycolicibacterium*, were created alongside an emended genus *Mycobacterium*. Upon publication in a validation list (18), the new names and circumscriptions were automatically applied by the National Center for Biotechnology Information (NCBI)’s Taxonomy Browser and affiliated gene/genomic databases. Because the name changes affected a number of clinically important pathogens, as well as a model organism in mycobacterial research (*Mycobacterium smegmatis*, new name *Mycolicibacterium smegmatis*), they did not reach consensus among the scientific community (19 – 21). Upon re-examination by combining a variety of approaches for defining genus boundaries, reconstitution of the original *Mycobacterium* taxon was subsequently proposed (12).

Another recent case in the *Mycobacteriales* is the creation of the genus *Prescottella* for the animal and zoonotic human pathogen *Rhodococcus equi* (22, 23). Originally described as *Corynebacterium equi* by Magnusson in 1923 (24), the genus *Rhodococcus* Zopf 1891 has been the stable home of *R. equi* since 1977 (25). However, echoing some low-resolution studies in the 1980s–1990s that failed to pinpoint its position in the *Nocardia*/*Rhodococcus* radiation (26 – 29), transfer of the species to a separate genus was (unsuccessfully) attempted in 2013, first as “*Prescottia equi,*” then “*Prescottella equi”* (https://lpsn.dsmz.de/genus/prescottella). Despite *R. equi* sharing evident physiological and genetic features with the other rhodococci (30), and its differentiation from the genus *Rhodococcus* being unsupported by compelling phylochemotaxonomic (31) and phylogenomic evidence (32), the transfer was proposed again and validly published in 2022 (22). The creation of the genu*s Prescottella* for the rhodococcal sublineage comprising *R. equi* renders the *Rhodococcus* genus paraphyletic, theoretically entailing the need to create new genera for every of the other major rhodococcal sublineages of equal rank (30).

In this work, the *Mycobacteriales* taxonomy and novel genus *Prescottella* were used as case study for genomics-based bacterial genus definition. We report herein a simple and reliable method for genus demarcation focused on a middle/high-rank (order) taxonomic context based on phylogenomic tree clustering and validation of relationships between taxa by network analysis using distance and genomic relatedness index matrices. Our approach seeks to maximize genus circumscription objectivity by uniform application of rank partitions across an entire taxonomic context encompassing a diversity of genera and families

## RESULTS

### *Mycobacteriales* core-genome phylogeny

Since a robust taxonomy is reliant on an accurate phylogeny, we began by performing a detailed reconstruction of the evolutionary relationships within the *Mycobacteriales*. A Maximum Likelihood (ML) tree was built from a supermatrix of 34 concatenated core-genome proteins from 156 species (see Materials and Methods and Table S1). Representatives of the diversity of all genera with available genomes in the NCBI database as of October 2022 were included in the analysis.

**Fig 1:**
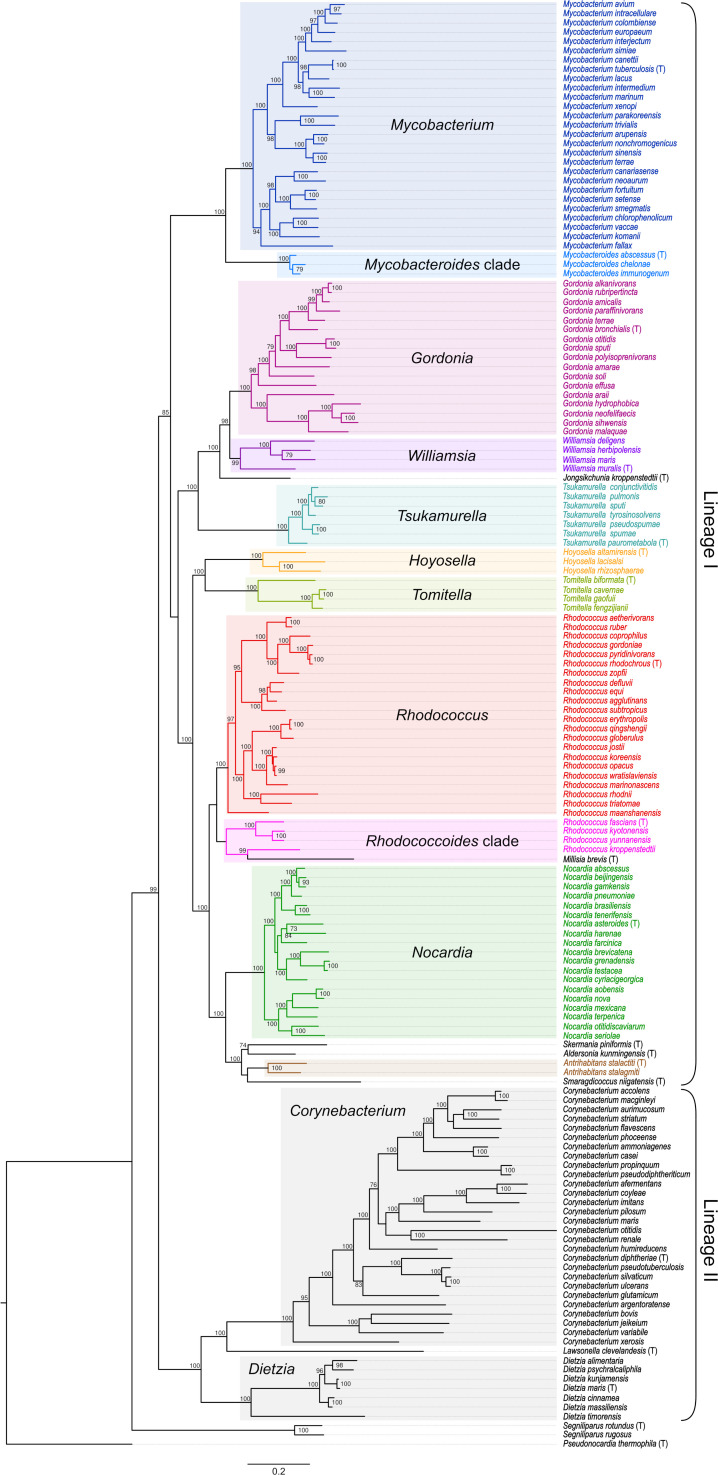
ML phylogenetic tree based on a concatenated alignment of 34 conserved proteins from 155 *Mycobacteriales* species plus *Pseudonocardia thermophila* DSM43832^T^ from the type genus of the order *Pseudonocardiales* as an outgroup (see Table S1). Bootstraps ≥70% are shown (10,000 replicates); see Fig. S1 for a bootstrap analysis represented as a consensus phylogenetic network. The tree contains representatives of the current 10 *Mycobacteriales* families, including the recently proposed *Hoyosellaceae* and *Tomitellaceae* (34). Three basal branches can be distinguished: the outermost corresponds to the *Segniliparaceae* family; the others are two main lines of descent comprising, respectively, the families *Mycobacteriaceae*, *Gordoniaceae*, *Tsukamurellaceae*, *Hoyosellaceae*, *Tomitellaceae*, and *Nocardiaceae* (herein called *Mycobacteriales* lineage I), and the families *Corynebacteriaceae*, *Lawsonellaceae*, and *Dietziaceae* (*Mycobacteriales* lineage II). These lineages have been validated by TreeCluster partitioning of the RED-normalized *Mycobacteriales* tree (Fig. 2) using a *t* = 1.03–1.10 cutoff range. Note the larger genetic distances and deeper branchings in lineage II, most obvious for the genus *Corynebacterium*, suggesting that the two major *Mycobacteriales* lines of descent have different evolutionary dynamics. Genera (including the mycobacterial *Mycobacteroides* clade/genus and the rhodococcal “fascians”/Rhodococcoides clade; see text) are color-coded (labels and shadowed boxes). Type species of each genus are indicated (T). Scale bar, amino acid substitutions per site. Tree plotted using FigTree v1.4.4 (http://tree.bio.ed.ac.uk/software/figtree/).

The ML tree shows that the *Mycobacteriales* diversity is distributed in two major lineages, here designated I and II (Fig. 1). Lineage I comprises the families *Mycobacteriaceae*, *Gordoniaceae*, *Tsukamurellaceae*, *Hoyosellaceae*, *Tomitellaceae*, and *Nocardiaceae*; lineage II, the families *Corynebacteriaceae*, *Lawsonellaceae* and *Dietziaceae*. An additional family *Segniliparaceae* with a single genus *Segniliparus* and only two species branched out near the root. The tree is congruent with previously reported actinobacterial phylogenies based on universal protein-coding gene markers (33, 34), with detailed resolution at genus level afforded by analytically determined *Mycobacteriales* core-genome phylomarkers (see Materials and Methods).

**Fig 2:**
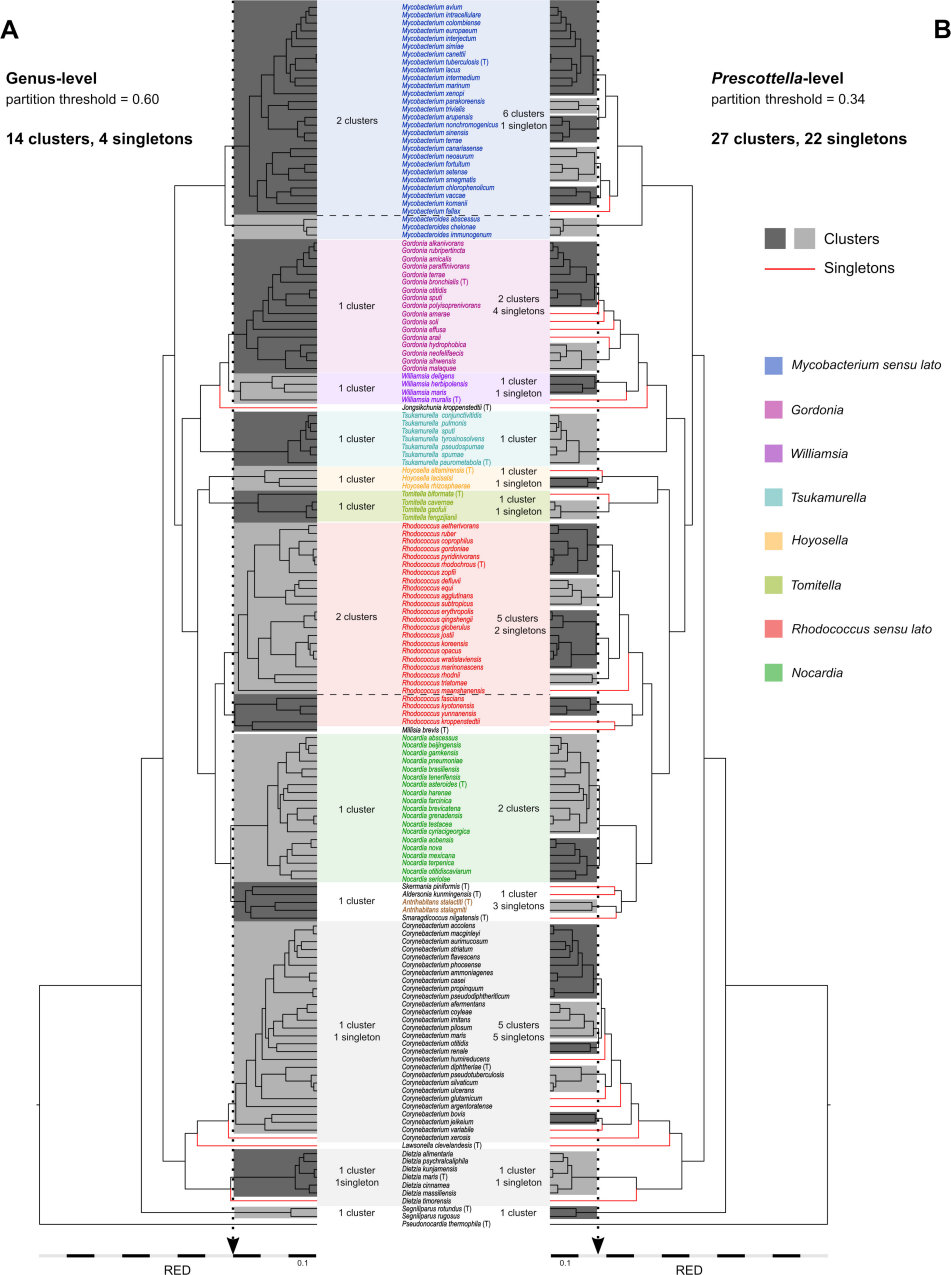
*Mycobacteriales* ML tree partitioning using TreeCluster (35) after relative evolutionary divergence (RED) branch length normalization (by assigning the values 0 and 1 to the root and each of the tree tips, respectively) (15). (**A**) Tree clustering using “classical” (pre-genomic; see text) genus-level partitioning threshold. (**B**) Tree partitioning using a threshold that isolates the *Prescottella* circumscription as an independent cluster (resulting in taxonomic atomization). The number of clusters and singletons generated in (**A**) and (**B**) are indicated. Clusters and singletons (each representing potential genera) are highlighted in the trees by alternate dark/light gray boxes and red lines, respectively. Genera demarcated at *t* = 0.60 are color coded according to Fig. 1. Trees plotted using FigTree v1.4.4 (http://tree.bio.ed.ac.uk/software/figtree/).

### Tree-based clustering

The hierarchical clustering in a phylogenetic tree is critical for taxonomic rank delineation but is not directly taken into account for genus demarcation based on genomic similarity indices (8, 13, 14, 36 – 39). We addressed this by using the TreeCluster program, which at a given operator-defined threshold groups the leaves into clusters based both on evolutionary distances (branch lengths) and phylogenetic relationships (tree topology) (35). Since the two major *Mycobacteriales* lineages I and II appeared to follow different evolutionary dynamics (Fig. 1), potentially posing constraints to the tree partitioning (35), prior to the TreeCluster analysis we scaled the ML tree according to relative evolutionary divergence (RED) using the PhyloRank package (15) (Fig. 2). A range of TreeCluster thresholds was tested using as a demarcation reference the major (multispecies) “classical” *Mycobacteriales* genera, that is, those defined according to “pre-genomic” taxonomic criteria (i.e. DNA-DNA hybridization, 16S rDNA/conserved housekeeping gene sequencing, molecular fingerprinting, chemotaxonomic and phenotypic markers, etc., and combinations thereof) (2, 4 – 11, 40). Namely: *Corynebacterium* Lehman and Neumann 1896 (Approved Lists 1980), *Dietzia* Rainey et al. 1995, *Gordonia* corrig. (*ex* Tsukamura 1971) Stackebrandt et al. 1989, *Hoyosella* Jurado et al. 2009, *Mycobacterium* Lehman and Neumann 1896 (Approved Lists 1980), *Nocardia* Trevisan 1889 (Approved Lists 1980), *Rhodococcus* Zopf 1891 (Approved Lists 1980), *Segniliparus* Buttler et al. 2005, *Tomitella* Katayama et al. 2010, *Tsukamurella* Collins et al. 1988, and *Williamsia* Kämpfer et al. 1999. The classical (pre-genomic) genera encapsulate the cumulative “conventional wisdom” and know-how from decades of collective practice by prokaryotic systematists and, hence, constitute an inescapable demarcation reference to achieve taxonomic continuity and stability. The overall robustness of the classical genera has been independently confirmed by different systematic studies based on phylogenomic markers and indices (6, 8, 13, 14, 36, 37).

A TreeCluster threshold *t* = 0.60 (see Materials and Methods) optimally recapitulated the established classical genera (Fig. 2A) while also addressing particular situations that required closer scrutiny (discussed below). Similar results were obtained for *Mycobacteriales* lineage I in the non-scaled ML tree but with a threshold range (0.64–0.70) instead of a single cutoff owing to the non-normalized phylogenetic depths across the different lines of descent (Fig. 1). Highlighting the different rate of evolution of lineage II relative to lineage I, application to the former of the same 0.64–0.70 cutoff range caused the atomization of the genus *Corynebacterium* into between 11 (0.64 cutoff) and 10 (0.70 cutoff) clusters and singletons (whereas it appeared as a single cluster plus a singleton in the distance-normalized tree).

Focusing on *Mycobacteriales* lineage I containing the genus *Rhodococcus*, the tree clustering revealed two specific cases where segregation of established genera could be justified. One is the basal rhodococcal branch containing *Rhodococcus fascians* NBRC 12155^T^, *Rhodococcus kropenstedtii* DSM 44908^T^, *Rhodococcus kyotonensis* JCM 23211^T^, and *Rhodococcus yunnanensis* NBRC 103083^T^ (Fig. 1 and 2A) (plus *Millisia brevis* NBRC 105863^T^, likely artifactually placed in this clade; see explanations in Fig. S1 and Fig. S5 legends). This earlier bifurcating “fascians” sublineage was previously identified as more distantly related to the rest of the rhodococci (30, 32), remained independent from the other rhodocci across a wide range of TreeCluster thresholds, and could warrant separate genus status.

The other case is the mycobacterial sublineage containing *Mycobacterium* (*Mycobacteroides*) *abscessus* ATCC 19977^T^, *Mycobacterium* (*Mycobacteroides*) *chelonae* CCUG 47445^T^, and *Mycobaterium* (*Mycobacteroides*) *immunogenum* CCUG 47286^T^ (henceforth the Mycobacteroides clade). This clade forms an early mycobacterial branching analogous to the rhodococcal “fascians” clade (Fig. 1 and 2A), suggesting that both should receive the same treatment. The more distant positioning of the Mycobacteroides clade was also noted in previous studies (17, 41), and its possible consideration as a separate genus was suggested by Meehan et al. in their recent re-examination of mycobacterial taxonomy (12).

### Network analysis of genomic indices

The tree clustering-based genus demarcation of *Mycobacteriales* lineage I encompassing the *Rhodococcus*/*Prescotella* case study was tested using different genome relatedness indices (GRI) reported to be discriminative for prokaryotic genus delineation: (i) average amino acid identity (AAI) of conserved protein-coding sequences (CDS) (6, 8, 36, 37); (ii) aligned fraction of orthologous genes (AF) (13, 42); and (iii) whole-genome average nucleotide identity of orthologous genes (gANI) (13, 42). A fourth metric, the percentage of conserved proteins (POCP) (14), was not used because we observed that genome relatedness inferences based on the latter could be affected by differences in genome size (Fig. S2).

Typically, GRI values from pairwise comparisons are represented in the form of frequency histograms, scatter/dot plots (12 – 14, 36, 38, 43), or similarity matrices visualized as heatmaps or dendrograms (39, 44 – 46). Here, instead, we took advantage of the powerful network analysis approach to spatially visualize the interrelationships between the genomes. We used BioLayout (47), an application that constructs three-dimensional networks from matrix inputs, in this case GRI pairwise comparison matrices. After import of the matrix file, a movable clustering threshold (ct) gradually splits the network into subnetworks representing potential taxonomic categories. At each selected ct cutoff, use of the Markov Clustering (MCL) algorithm embedded in BioLayout non-subjectively divides the network graph into discrete “similarity” modules (47), which can then cross-checked against the (established or proposed) taxons. Unlike classical pairwise analyses, the network approach uses multiway comparisons and thus captures higher-order similarity linkages, allowing the detailed graphical exploration of the relatedness between multiple genomes in a visual and intuitive manner. For comparative purposes and as an additional reference, network graphs were also constructed using an ML distance matrix from the Fig. 1 dataset.

**Fig 3:**
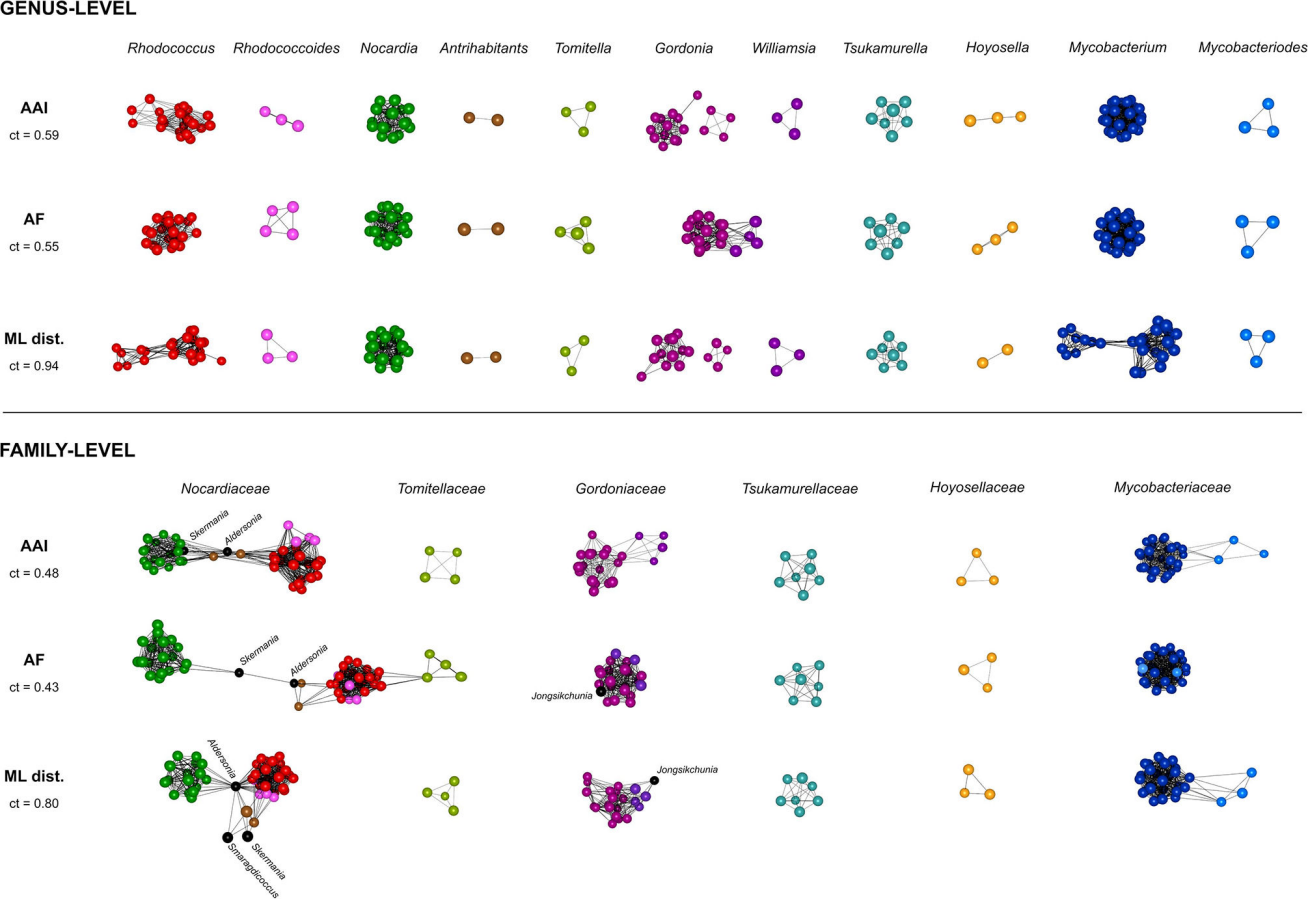
Phylogenomic 3D network analysis based on GRI (AAI, AF) and ML distance (ML dist.) matrices. *Mycobacteriales* species analyzed are those of lineage I shown in Fig. 1 (117 genomes). Two different network graph clustering thresholds (ct) were used for each genome-wide relatedness metric: a genus-level ct which individualized the “classical” (pre-genomic) genera (see text) into discrete clusters/subnetworks; and a less stringent family-level ct which formed discrete clusters/subnetworks roughly corresponding to the *Mycobacteriales* families. Each node represents a genome, interconnecting edges show relatedness between nodes above the ct; the closer the nodes sit in the network, the stronger the genomes are related. Genera are color coded according to Fig. 1. See Movies S1 and S2 for *Mycobacteriales* lineage I genus-level and family-level 3D network animations. Note that at the genus-level ct in both the AAI and ML distance network graphs, the genus *Gordonia* is split into two subnetworks. This reflects that the two lines of descent of the *Gordonia* radiation (see trees in Fig. 1 and 2) could potentially be considered separate genera. The monospecific genera *Aldersonia*, *Jongsikchunia*, *Millisia*, *Skermania*, and *Smaragdicoccus* are singletons and thus are not shown in the genus-level panel but may appear in the family-level network graphs.

As shown in Fig. 3 and Fig. S3, there was a close concordance between the genus-level tree-based clusterings and the GRI/ML distance-based network clusterings, with only minor differences in the topology of the groupings. Virtually all the demarcated genera by tree clustering (Fig. 2A) were confirmed as distinct, separate entities (i.e. discrete subnetworks) upon uniform application of network graph ct cutoffs across all GRI matrix data sets (Fig. 3 upper panel). This extends to both the rhodococcal “fascians” and the mycobacterial Mycobacteroides clades, adding strong objective support to their classification as independent genera. Furthermore, in the AF and gANI network analyses, the *Williamsia* and *Gordonia* genera still remained interconnected in the same subnetwork at ct values where the “fascians” and Mycobacteroides clades already appeared as separate subnetworks, like most other *Mycobacteriales* lineage I genera (Fig. 3; Fig. S3).

Network graphs obtained by lowering the ct cutoffs also reliably recapitulated the family structure of *Mycobacteriales* lineage I (Fig. 3 lower panel, Fig. S3 upper panel), highlighting the general usefulness of the network analysis approach as a taxonomic rank demarcation tool (see also Movies S3 to S7).

### Focused analysis of the *Nocardiaceae*

Additional analyses were performed with the genomes of all *Nocardiaceae* species available at NCBI at the time of the study (Table S2, see Materials and Methods) to further assess the genus demarcations and accurately define the taxonomic circumscriptions within the *Rhodococcus* genus (*sensu lato*) case study. To capture the breadth of rhodococcal diversity, we included representatives of all NCBI genome entries labeled as “uncharacterized *Rhodococcus* spp.” An average nucleotide identity (ANI) ≥95% filter [the cutoff for species delineation (4, 8, 48)] was applied to select one genome per terminal branching within each major *Rhodococcus* spp. sub-branch as displayed in the genomic Blast dendrogram (https://www.ncbi.nlm.nih.gov/genome/?term=txid192944, accessed November 2022).

**Fig 4:**
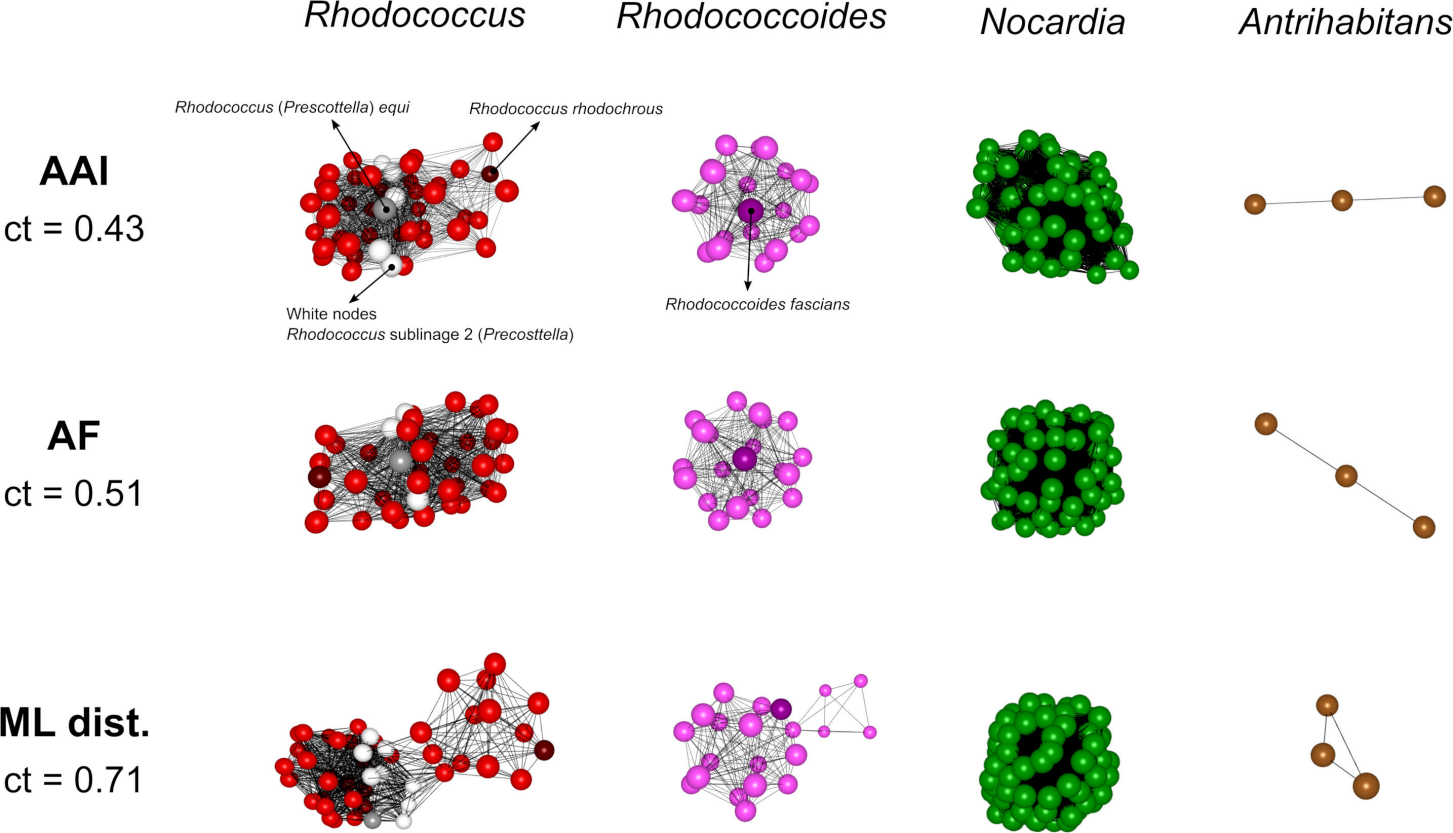
Phylogenomic 3D network analysis of the *Nocardiaceae* genomes shown in Fig. 4 tree (Table S2) using genus-level network graph clustering threshold (ct). See explanations in the legend to Fig. 3. The network graphs show that the *Prescottella* members are deeply embedded in the *Rhodococcus* (*sensu stricto*) cluster [red nodes, those of sublineage no. 2 (*Prescottella*) in white]. Indicated are the *Rhodococcus* type species, *R. rhodochrous* (dark red), and the *Prescottella* type species, *R. equi* (in gray). Note that at the genus-level ct, the “fascians” clade forms a discrete subnetwork, supporting its consideration as a novel genus (with the proposed name *Rhodococcoides*, see text; type species *Rhodococcoides fascians* in darker pink shade). The monospecific genera *Aldersonia*, *Millisia*, *Skermania,* and *Smaragdicoccus* are singletons and do not appear in the network grap. See Fig. S6 for corresponding family-level graph clustering analyses of the *Nocardiaceae*.

An ML tree constructed with the *Nocardiaceae* genome assemblies reflected the typical internal diversification of bacterial genera into a number of well-defined monophyletic sublineages (Fig. S4). Although including many more genomes per subclade (and concatenated phylomarkers in the phylogenetic analysis, 53 vs 34), the branching topology was perfectly congruent with the *Mycobacteriales* tree, supporting the overall robustness of our ML phylogenies (Fig. 1; Fig. S4A). Specifically, the *Nocardiaceae* tree corroborated the evident early separation of the “fascians” clade from the rhodococcal line of descent in a well-supported branch (Fig. S4A). The “fascians” clade again appeared as a discrete grouping in both the tree clustering and the ML-/GRI-based network analyses without corresponding fragmentation of the main *Nocardiacea*e genera (Fig. 4 and Fig. S4B). Overall, these data lend strong support to the adequacy of elevating the “fascians” clade to genus status. All the other rhodococcal species, including those reclassified into the recently proposed genus *Prescottella*, formed together a well-defined monophyletic grouping with genus rank (Fig. 4; Fig. S4).

Of note, application of the genus-level cutoffs in both the TreeCluster and network analyses clearly placed *Spelaeibacter* (formerly *Rhodococcus*) *cavernicola* C1-24^T^ within the *Antrihabitants* genus (Fig. 4; Fig. S4B). Our data, therefore, do not support the recent creation within the *Antrihabitans* radiation of the genus *Spelaeibacter*, with *Spelaeibacter cavernicola* as its sole species (49). Additional conclusions from the *Nocardiaceae* phylogenomic analysis can be found in the Supplemental Text.

### *Prescottella*-level demarcation leads to *Mycobacteriales* taxonomic atomization

**Fig 5:**
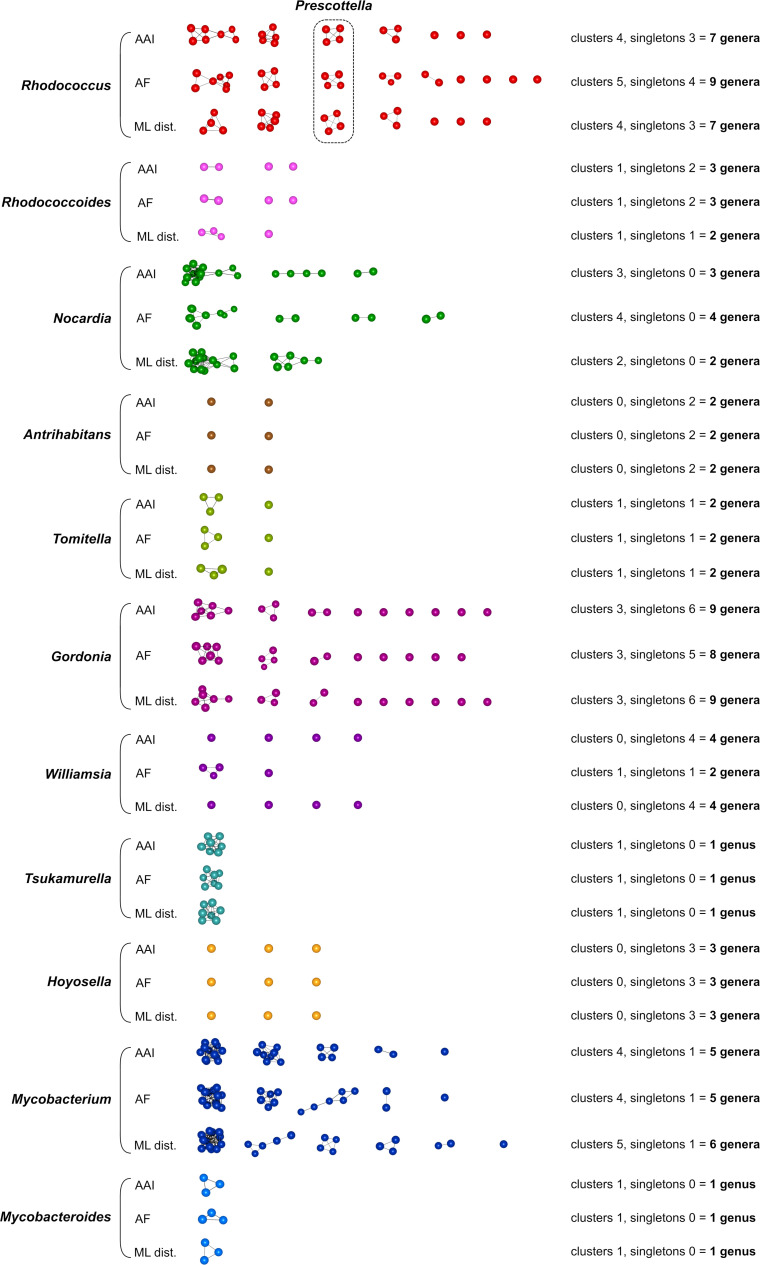
Generalized taxonomic atomization resulting from application of *Prescottella*-level taxon demarcation threshold. Same *Mycobacteriales* lineage I taxonomic network analysis as in Fig. 3 but using a network graph clustering threshold (ct) that individualizes the rhodococcal *Prescottella* circumscription in an independent subnetwork. For interpretation, see explanations in the legend to Fig. 3. The number of potential genera that result from splitting the network graph in *Prescottella* genus-level clusters is indicated on the right.

We finally examined the impact that the creation of the genus *Prescottella* (22) potentially has on the *Mycobacteriales* taxonomy by repeating our analyses using the tree partitioning and network graph thresholds that isolated the *Prescottella* circumscription (*R. equi*, *R. defluvii*, *R. subtropicus*, *R. agglutinans*; Fig. 1) in a separate cluster/subnetwork. This was achieved with a tree-clustering threshold *t* around 0.34 and resulted in an increase from 14 clusters and 4 singletons (Fig. 2A) to 27 clusters and 22 singletons (Fig. 2B), representing a total of 49 potential genera equivalent in rank to *Prescottella* (an ≈threefold increase in the *Mycobacteriales* genus content). In *Mycobacteriales* lineage I, the rhodococci (including the “fascians” clade) and the mycobacteria are fragmented into seven potential new genera each, and *Gordonia* into six potential genera (Fig. 2B). In the rhodococci, the fragmentation corresponds to the five previously observed main intra-generic genomic groups/sublineages (32, 39), plus two singletons (*Rhodococcus kroppenstedtii* DSM 44908^T^, *Rhodococcus maanshanensis* NBRC 100610^T^); for *Mycobacterium*, it roughly corresponds to the five genera that resulted from the recent subdivision of the genus by Gupta et al. (17), plus a singleton (*Mycobacterium fallax* JCM 6405^T^). The genera *Nocardia*, *Williamsia,* and *Tomitella* were also split to different extents (Fig. 2B). In *Mycobacteriales* lineage II, *Corynebacterium* would be subdivided into 10 potential new genera. The GRI/ML distance-based network analyses mirrored the tree-based clustering (Fig. 5). At ct cutoffs within the *Prescottella-* (and mycobacterial five-genus)-level demarcation range, the number of independent subnetworks increases from the original 10–12 clusters and 5–10 singletons (Fig. 3A) to 18–21 clusters and 26–32 singletons (≈twofold to threefold increment in potential genera) (Fig. 5).

## DISCUSSION

The advent of genomics and the development of genome-wide similarity metrics have been paradoxically accompanied by a blurring of the prokaryotic genus boundaries, with an increasing trend towards genus rank fragmentation (17, 22, 38, 43, 50, 51). Because the inconsistent application of genomics-based demarcation criteria may lead to taxonomic and nomenclatural instability, it is critical to tackle this emerging problem by establishing well-defined and easily applicable standardized methods for genus delineation.

### Consistent, non-subjective genus definition

Toward this goal, here we report a practical and straightforward approach that minimizes subjectivity in genus demarcation based on distance-normalized phylogenomic tree clustering and taxonomic cluster verification by network analysis of genomic relatedness indices (GRI). The AAI, AF, and gANI metrics (13, 37, 42), as well as an ML distance score, all afforded consistent genus discrimination in the network analyses. However, we found gANI (42) to be less useful due to its narrower ct dynamic range for genus delimitation (see Fig. S3 legend).

The proposed genus delineation approach rests on three key guiding principles: (i) analytical objectivity, by uniform application of the same demarcation criteria and thresholds across the relevant taxonomic context; (ii) consideration of the cumulative taxonomic knowledge, by taking the classical (pre-GRI-based) genera circumscriptions as demarcation reference; and (iii) compliance with the Prokaryotic code, by aiming at the stability of names and avoiding the useless creation thereof (3). Point (i) is the cornerstone for an unbiased genus definition. The taxonomic context should necessarily comprise a range of established genera as a reference—ideally from a number of different families—, that is, order level, as illustrated here with the *Mycobacteriales*.

**Fig 6:**
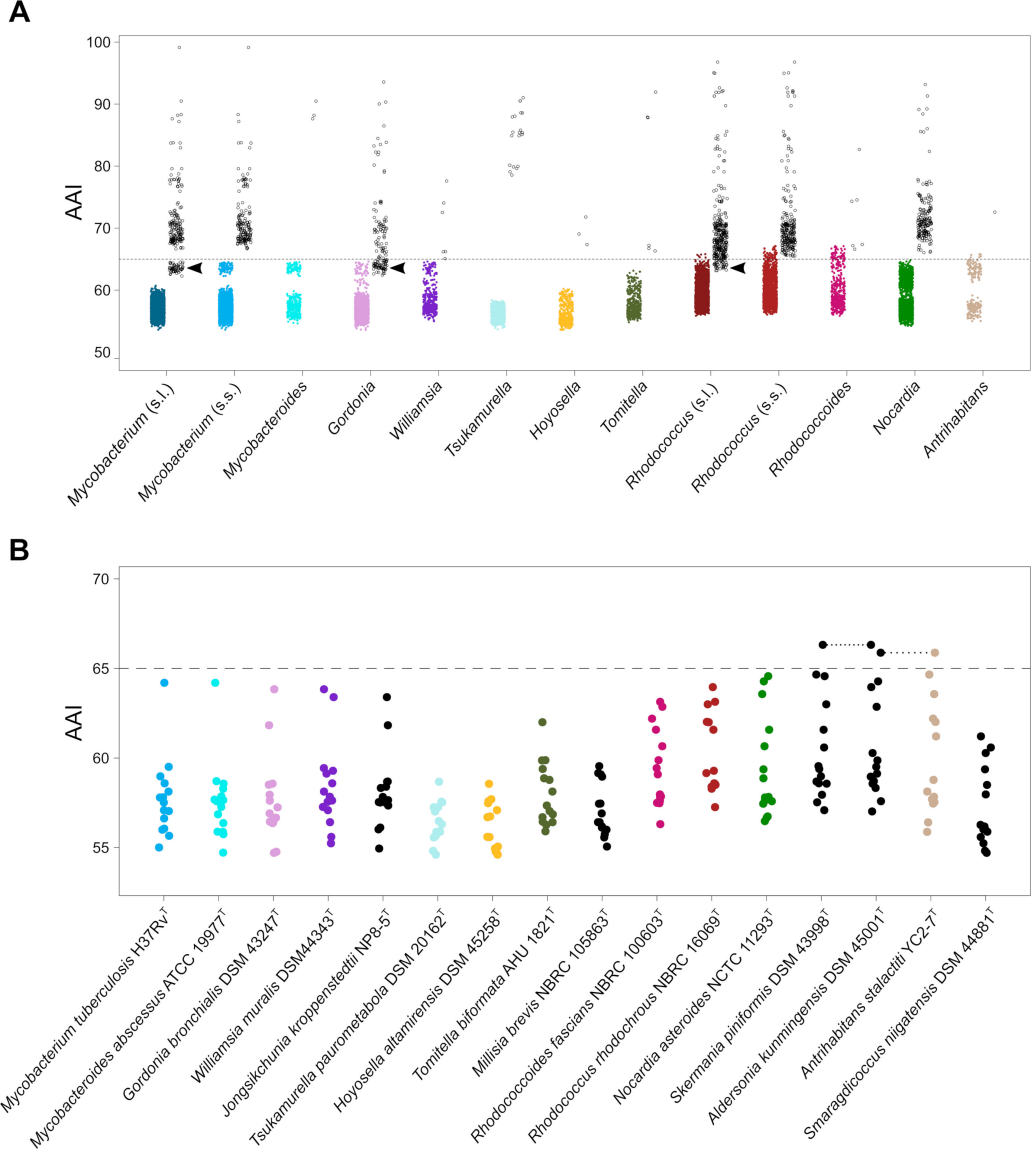
Scatter plots of *Mycobacteriales* (lineage I) AAI scores. The horizontal dashed line indicates the standard AAI genus demarcation boundary of 65% (8, 36, 37). (**A**) Distribution of inter-genus (solid dots, colored according to Fig. 1 coding) and intra-genus (empty dots) AAI values. Analysis based on a total of 16,529 pairwise comparisons. Note that most inter-genus values are below the AAI 65% genus demarcation line while the intra-genus AAI scores are >65% (*P* < 2.2 × 10^–16^, one-sample Wilcoxon test). The only exceptions are indicated by arrowheads and correspond to *Mycobacterium* (*sensu lato*), *Rhodococcus* (*sensu lato*), and *Gordonia*, indicating that their diversity includes members of other genera. For *Mycobacterium* and *Rhodococcus* "*sensu lato*" (indicated s.l.), this leakage into the <65% zone is resolved when the Mycobacteroides and “fascians” (*Rhodococcoides*) clades are segregated as independent genera from *Mycobacterium* and *Rhodococcus* “*sensu stricto*” (indicated s.s.), respectively. The case of *Gordonia* requires further studies to determine whether each of its two major sublineages (Fig. 1 and 2) warrant genus rank, as suggested by our AAI and ML distance network analyses (Fig. 3). (**B**) Distribution of AAI scores for pairwise comparisons between the type strain of each genus. Note that all AAI scores are <65%, including those for *Mycobacteroides abscessus* ATCC 19977^T^ and *Rhodococcoides* (*Rhodococcus*) *fascians* NBRC 100603^T^ (the type strain of the novel genus *Rhodococcoides* proposed here) (*P* < 2.2 × 10^–16^, one-sample Wilcoxon test). The only exceptions are the AAI scores between *Aldersonia kunmingensis* DSM 45001^T^ and *Skermania piniformis* DSM 43998^T^ (66.32%) or *Antrihabitans stalactiti* YC2-7^T^ (65.88%), reflecting a potentially closer relationship between these poorly represented genera.

Our genus demarcations are in almost perfect concordance with the genome-aggregate average AAI value of 65%, recognized as a robust genomic discreteness standard for genus definition in both natural isolates and metagenomic sequences (6, 8). Except for *Gordonia* (discussed in Fig. 3 and Fig. 6A), all the demarcated genera consistently had internal AAI scores above the 65% boundary, while the corresponding inter-generic values were below this cutoff. This was also true for the type species of all the genera circumscriptions (Fig. 6). Emphasizing the robustness of the proposed method, our taxonomic conclusions are also in general agreement with the Genome Taxonomy Database (GTDB) classification based on normalized evolutionary distances (https://gtdb.ecogenomic.org/) (15). The GTDB (release 207 April 2022) considers a main rhodococcal genus, which coincides with our *Rhodococcus* “*sensu stricto*” (including the *Prescottella* circumscription), plus two additional genus-rank taxa (designated "a" and "b") which correspond to the two sublineages within the “fascians” clade (see Fig. S4). GTDB also considers the mycobacteriae to be a single genus (for which, based on our data, we propose the refinement of classifying its Mycobacteroides sublineage as an independent genus).

### Taxonomic fragmentation caused by shifting the genus boundary to sublineage level

Raising the genus-definition threshold to the *Prescottella* (or mycobacterial five-genus) level broke up the *Mycobacteriales* into multiple potential new genera (Fig. 2B and 5). Specifically, the rhodococcal and mycobacterial diversity was split into subclusters/subnetworks which roughly corresponded to the internal sublineages of the classical (*sensu lato*) *Rhodococcus* and *Mycobacterium* genera—for each of which genus status was implied or claimed by Sangal et al. (22) and Gupta et al. (17). The strong parallelism between the *Rhodococcus* and *Mycobacterium* cases is striking, highlighting that the recent splits in these genera (and generally, the current genus over-splitting trend) have a common cause: the elevation of the sublineages inherent to the internal diversity of prokaryotic genera to genus rank due to the use of arbitrary genomics-based demarcation boundaries.

**Fig 7:**
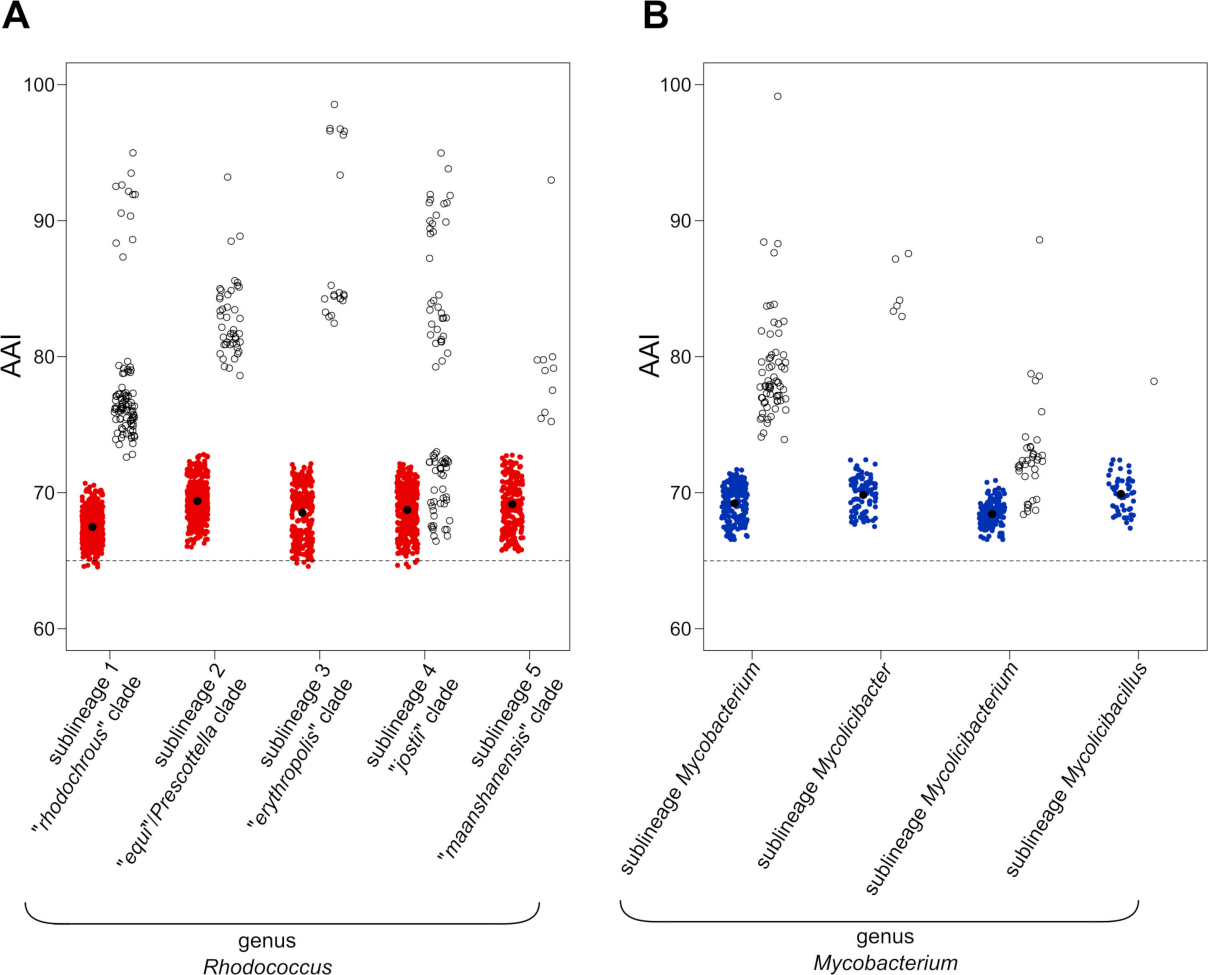
Scatter plots of *Rhodococcus* (*sensu stricto*) and *Mycobacterium* (*sensu stricto*) AAI scores. The horizontal dashed line indicates the standard AAI genus demarcation boundary of 65% (6, 8, 37). Inter-sublineage comparisons are represented as solid dots with color-coding as per Fig. 1; the solid black dot in each column is the mean AAI value. Intra-sublineage comparisons are represented as empty dots. (**A**) *Rhodococcus* (*sensu stricto*) pairwise comparisons (*n* = 2,059, 49 genomes). (**B**) *Mycobacterium* (*sensu stricto*) pairwise comparisons (*n* = 593, 27 genomes). Note that virtually all inter-sublineage AAI scores are above the 65% genus definition boundary (*P* < 2.2 × 10^–16^, sublineage *Mycolicibacillus P* < 3.89 × 10^–10^; one-sample Wilcoxon test). The mean inter-sublineage AAI values between the five *Rhodococcus* (*sensu stricto*) and between the four *Mycobacterium* (*sensu stricto*) sublineages (68.56 ± 1.69 and 69.14 ± 1.32, respectively) are significantly different from the mean AAI values between the genera *Rhodococcoides* and *Mycobacteroides* and the *Rhodococcus* (*sensu stricto*) or *Mycobacterium* (*sensu stricto*) sublineages (64.65 ± 1.16 and 63.63 ± 0.52, respectively) (*P* < 2.2 × 10^–16^, two-sample Wilcoxon test).

Indeed, the recent reclassification by Sangal et al. of the rhodococcal sublineage containing *R. equi* DSM 20307^T^ (=ATCC 6939^T^) into a novel genus *Prescottella* used an AAI genus demarcation threshold of 74%–75% instead of 65% (22). The higher AAI cutoff was justified based on work that led to the split of the flavobacterial genus *Chryseobacterium* (43), and the latter in turn by earlier work by Sangal et al. (39) (supporting the subdivision of the genus *Rhodococcus*) and Gupta et al. (17) (supporting the subdivision of the genus *Mycobacterium*). Similar to other recent studies proposing the fragmentation of bacterial genera (38, 51), Sangal et al. (22) also considered POCP genus threshold values significantly higher than the 50% cutoff originally proposed by those who developed this GRI (14). The use of higher GRI cutoffs has been justified, for example, with the argument that prokaryotic taxa exhibit a continuum of AAI values within a given non-overlapping range (37), and thus, an AAI gradient rather than a discrete boundary should be employed (38). This probably reflects a misunderstanding, because while AAI values between members of the same genus may range between 65% and 95% (37) (see also Fig. 6A), a 65% cutoff has been recognized as a universal indicator of affiliation to a different genus (8, 36, 37). Another probable reason behind the higher GRI scores considered in the “genus-splitting” studies is a narrow taxonomic context for the pairwise comparisons, restricted to a phylogenetically closely related diversity (likely representing intra-generic sublineage groupings, as opposed to the wider—order level—taxonomic context of our analyses). As shown in Fig. 7, the members of each of the rhodococcal and mycobacterial sublineages —which according to Sangal et al. (22) and Gupta et al. (17) criteria should have genus status— have mean inter-sublineage AAI values of 68.4 ± 0.5 and 70.2 ± 0.7, respectively, well above the recognized 65% genus demarcation cutoff.

### Conclusions

Implementation of our phylogenomic approach highlights the novel genera recently created within the rhodococcal (22), mycobacterial (17), and *Antrihabitans* (49) radiations as paradigmatic examples of an emerging genus fragmentation trend (17, 22, 38, 43, 50, 51). Our data indicate that these genus splits are not justified. Generalized use of genus-oversplitting demarcation criteria may lead to a very significant atomization of the prokaryotic taxonomy, as illustrated here with the *Mycobacteriales*.

Although our analyses do not support the creation of the *Prescottella* genus (and consequent subdivision of *Rhodococcus* in at least six different genera) or the split of *Mycobacterium* into five genera, they identify two early-diverging branches in each of the corresponding bacterial groups that would warrant genus status: (i) the clade containing *R. fascians* NBRC 12155^T^, for which we propose the genus name *Rhodococcoides* gen. nov*.*; and (ii) the clade containing *M. abscessus* ATCC 19977^T^, previously designated *Mycobacteroides* by Gupta et al. (17).

In summary, our phylogenomic analyses result in: (a) confirmation of the “classical” (pre-genomic) *Mycobacteriales* genera, with the caveat that *Gordonia* may comprise more than one genus (discussed in Fig. 6A legend); (b) emendation of *Rhodococcus* Zopf 1891 (Approved Lists 1980), with inclusion of the *Prescottella* circumscription and reclassification of the “fascians” clade in a new genus *Rhodococcoides*; (c) emendation of *Mycobacterium* Lehmann and Neumann 1896 (Approved Lists 1980) as recently emended by Meehan et al. 2021 (12), with transfer of the Mycobacteroides (17) clade to an independent genus; (d) confirmation of the recently defined *Jongsikchunia* Nouioui et al. 2018 (52) and *Antrihabitans* Lee et al. 2020 (53), with transfer of *Spelaeibacter* (*Rhodococcus*) cavernicola C1-24^T^ to the latter as *Antrihabitans cavernicola* comb. nov. Determining the exact affiliation of the genera *Antrihabitans*, *Aldersonia*, *Millisia,* and *Smaragdicoccus* within the rhodococcal/nocardial line of descent will require the analysis of a larger diversity of isolates not yet available.

Finally, the different evolutionary dynamics observed for the *Mycobacteriales* lineages I and II, together with their clear demarcation in the tree (Fig. 1), support a case for formally assigning them to distinct suborders. We propose for these, respectively, the names *Mycobacteriineae* subord. nov., and *Corynebacteriineae* [reusing a name originally proposed by Stackebrandt et al. (54)].

## TAXONOMIC DESCRIPTIONS

### *Rhodococcoides* gen. nov.

*Rhodococcoides* (Rho.do.coc.co’i.des. N.L. neut. n. *Rhodococcus*, a bacterial genus; L. neut. suff. *-oides*, resembling; N.L. neut. n *Rhodococcoides*, a genus resembling *Rhodococcus*). Members of this genus share many characteristics of the genus *Rhodococcus*. They are Gram-positive, non-motile, non-spore-forming, strictly aerobic, and catalase positive. Cell morphology from coccoid to short rods or hyphae that fragment into rods/coccobacilli. They form colonies from pale yellow to orange or orange-red pigmented and contain mycolic acids as a salient chemotaxonomic property. The genus contains the species *R. corynebacteroides*, *R. fascians*, *R. kroppenstedtii*, *R. kyotonensis*, *R. trifolii*, and *R. yunnanensis*. They are found in soil, as culture contaminants, or epiphytic, with some (*R. fascians*) causing plant pathogenesis. Niche adaptation in the plant pathogenic *R. fascians* is mediated by a virulence plasmid like in *R. equi*. Genus comprises two main sublineages, “a” (represented by *R. fascians*) and “b” (represented by *R. corynebacteroides*). Genomes have a G + C content ranging from 64.5% to 70.5% and genome size ranging from 3.9 to 6.4 Mpb, with sublineage “a” tending to have lower G + C values (64%–65%) and larger genomes (5.7–6.4 Mbp). Members of the genus can be distinguished from *Rhodococcus* and other genera currently classified in the family *Nocardiaceae* by relative evolutionary divergence (RED)-normalized tree clustering as well as genome-relatedness index (GRI)- and maximum likelihood distance-based network analysis. Using this approach, they are individualized in a discrete cluster or subnetwork when applying clustering or network graph cutoffs that form taxonomic units consistent with the classical (pre-genomic) *Mycobacteriales* genera. All *Rhodococcoides* members typically have an AAI score ≤64 with *R. rhodochrous* NBRC 16069^T^, the type species of the genus *Rhodococcus*. Type species: *Rhodococcoides fascians.*

### *Rhodococcoides corynebacterioides* comb. nov.

*Rhodococcoides corynebacterioides* (co.ry.ne.bac.te.ri.oi’des. N.L. neut. n. *Corynebacterium* bacterial genus name; Gr. suffix -oides similar to; N.L. adj. *corynebacterioides* similar to *Corynebacterium*). Basonym: *Rhodococcus corynebacterioides* (Serrano et al. 1972) Yassin and Schaal 2005. The description of this taxon is given in reference (55). The type strain has a G + C content of 70.5% and a genome size of 3.9 Mbp. Type strain: DSM 20151^T^ (= ATCC 14898^T^ = CCUG 37877^T^ = CIP 104510^T^ = IFO 14404^T^ = JCM 3376^T^ = JCM 3391^T^ = NBRC 14404^T^ = NRRL B-24037^T^).

### *Rhodococcoides fascians* comb. nov.

*Rhodococcoides fascians* (fa.sci.ans. L. part. adj. *fascians*, binding together, bundling). Basonym: *Rhodococcus fascians* (Tilford 1936) Goodfellow 1984; “*Phytomonas fascians*” Tilford 1936. The description of this taxon is as given by Goodfellow (56). The type strain has a G + C content of 64.5% and a genome size of 5.8 Mbps. Type strain: DSM 20669^T^ (= ATCC 12974^T^ = CFBP 2401^T^ = CIP 104713^T^ = ICMP 5833^T^ = IFO 12155^T^ = JCM 10002^T^ = LMG 3623^T^ = NBRC 12155^T^ = NCPPB 3067^T^ = NRRL B-16937^T^ = VKM Ac-1462^T^).

### *Rhodococcoides kroppenstedtii* comb. nov.

*Rhodococcoides kroppenstedtii* (krop.pen.stedt’i.i. N.L. gen. masc. n. *kroppenstedtii*, of Kroppenstedt, to honor Reiner Michael Kroppenstedt, a German microbiologist, for his significant contributions to the taxonomy of actinomycetes). Basonym: *Rhodococcus kroppenstedtii* Mayilraj et al. 2006. The description of this taxon is as given by Mayilraj et al. (57). The type strain has a G + C content of 70.0% and a genome size of 4.1 Mbp. Type strain: K07-23^T^ (= DSM 44908^T^ = JCM 13011^T^ = MTCC 6634^T^ = NBRC 103113^T^).

### *Rhodococcoides kyotonense* comb. nov.

*Rhodococcoides kyotonense* (kyo.to.nen’se. N.L. neut. adj. *kyotonens*e, pertaining to Kyoto, the source of soil from which the organism was isolated). Basonym: *Rhodococcus kyotonensis* Li et al. 2007. The description of this taxon is as given by Li et al. (58). The type strain has a G + C content of 64.0% and a genome size of 6.3 Mbp. Type strain: DS472^T^ (= CCTCC AB 206088^T^ = DSM 45159^T^ = IAM 15415^T^ = JCM 23211^T^).

### *Rhodococcoides trifolii* comb. nov.

*Rhodococcoides trifolii* (tri.fo’li.i. L. neut. n. *trifolium*, three-leaved grass, trefoil and also a scientific genus name [*Trifolium*]; N.L. gen. neut. n. *trifolii*, of trefoil, isolated from *Trifolium repens*). Basonym: *Rhodococcus trifolii* Kämpfer et al. 2013. The description of this taxon is as given by Kämpfer et al. (59). The type strain has a G + C content of 65.5% and a genome size of 5.3 Mbp. Type strain: T8^T^ (= CCM 7905^T^ = DSM 45580^T^ = LMG 26204^T^).

### *Rhodococcoides yunnanens*e comb. nov.

*Rhodococcoides yunnanense* (yun.nan.en’se. N.L. neut. adj. *yunnanense*, of or pertaining to Yunnan, a province of south-west China). Basonym: *Rhodococcus yunnanensis* Zhang et al. 2005. The description of this taxon is as given by Zhang et al. (60). The type strain has a G + C content of 64.0% and a genome size of 6.4 Mbp. Type strain: YIM 70056^T^ (= CCTCC AA 204007^T^ = DSM 44837^T^ = JCM 13366^T^ = KCTC 19021^T^ = NBRC 103083^T^ = NBRC 103115^T^).

### *Antrihabitans cavernicola* comb. nov.

*Antrihabitans cavernicola* (ca.ver.ni’co.la. L. fem. n. *caverna*, a cave; L. masc./fem. n. suff. *-cola*, dweller; from L. masc./fem. n. *incola*, inhabitant, dweller; N.L. masc./fem. n. *cavernicola*, a cave dweller, referring to the site where the type strain was isolated). Basonym: *Rhodococcus cavernicola* Lee et al. 2020. Synonym: *Spelaeibacter cavernicola* (Lee et al. 2020) Kim et al. 2022. The description of this taxon is as given by Kim et al. (49). The type strain has a G + C content of 64.5% and a genome size of 5.7 Mbp. Type strain: C1-24^T^ (= DSM 109484^T^ = KACC 19964^T^).

### *Mycobacteriineae* subord. nov.

*Mycobacteriineae* (My.co.bac.te.ri.i’ne.ae. N.L. neut. n. *Mycobacterium*, type genus of the suborder; suff. -*ineae* ending to denote a suborder; N.L. fem. pl. n. *Mycobacteriineae*, the *Mycobacterium* suborder). The member families of this suborder (*Mycobacteriaceae*, *Nocardiaceae*, *Gordoniaceae*, *Tsukamurellaceae*, *Hoyosellaceae*, and *Tomitellaceae*) can be differentiated from other families of the order *Mycobacteriales* (*Corynebacterium*, *Lawsonellaceae,* and *Dietzia*) by phylogenomic analysis. In a core-genome phylogenetic tree, they constitute one of the two major lines of descent of the *Mycobacteriales*, the other being the *Corynebacteriineae* (with the families *Corynebacteriaceae*, *Dietziaceae,* and *Lawsonellaceae*). The estimated average genome size of the suborder is 5.7 ± 1.4 and G + C content is 68 ± 2.2. The type genus of the suborder is *Mycobacterium* Lehmann and Neumann 1896 [Approved Lists 1980 (55)].

### *Rhodococccus* Zopf 1891 (Approved Lists 1980) emend.

The description of this genus is as given in Goodfellow and Alderson (25) and Jones et al. (27), excluding the species in the most basal branch of the rhodococcal radiation (*R. corynebacterioides*, *R. fascians*, *R. kroppenstedtii*, *R. kyotonens*e, *R. trifolii*, and *R. yunnanense*), which are transferred to *Rhodococcoides* gen. nov., and comprising the circumscription of the genus *Prescottella* Sangal et al. 2022 (22), which is reclassified into the genus *Rhodococcus*. Core-genome phylogenies as well as RED-normalized tree clustering and GRI- and maximum likelihood distance-based network analyses all show that the *Prescottella* species belong to an internal lineage of the genus *Rhodococcus*. This is also supported by a genome-wide average AAI value ≥68% in pairwise comparisons with the *Rhodococcus* type species. The genus includes the species *R. aerolatus*, *R. aetherivorans*, *R. agglutinans*, *R. antarcticus*, *R. antrifimi*, *R. artemisiae*, *R. canchipurensis*, *R. cercidiphylli*, *R. chubuensis*, *R. coprophilus*, *R. defluvii*, *R. electrodiphilus*, *R. equi*, *R. erythropolis*, *R. gannanensis*, *R. globerulus*, *R. gordoniae*, *R. humicola*, *R. jostii*, *R. koreensis*, *R. lactis*, *R. maanshanensis*, *R. marinonascens*, *R. nanhaiensis*, *R. obuensis*, *R. olei*, *R. opacus*, *R. oryzae*, *R. oxybenzonivorans*, *R. pedocola*, *R. phenolicus*, *R. pseudokoreensis*, *R. pyridinivorans*, *R. rhodnii*, *R. rhodochrous*, *R. ruber*, *R. soli*, *R. sovatensis*, *R. spelaei*, *R. spongiicola*, *R. subtropicus, R. triatomae*, *R. tukisamuensis*, *R. wratislaviensis*, *R. xishaensis*, *R. yananensis*, and *R. zopfii*. The type species of the genus is *Rhodococcus rhodochrous* (Zopf 1891) Tsukamura 1974 [Approved Lists 1980 (55)].

### *Mycobacteroides* Gupta *et al*. 2018 emend.

The description of the genus *Mycobacteroides* is as given by Gupta et al. (17). Members of this genus can be distinguished based on core-genome phylogenetic analyses where they form the most basal branch of the *Mycobacteriaceae* family. They can be also differentiated by means of RED-normalized tree clustering as well as GRI- and maximum likelihood distance-based network analysis. Using this approach, they are individualized in a discrete cluster or subnetwork when applying clustering or network graph cutoffs that form taxonomic units consistent with the classical (pre-genomic) *Mycobacteriales* genera. *Mycobacteroides* members typically have an AAI score ≤64.5 with *Mycobacterium tuberculosis* H37Rv^T^, the type species of the genus *Mycobacterium*. The type species of the genus is *Mycobacteroides abcessus.*

### *Mycobacterium* Lehmann and Neumann 1896 emend. Meehan *et al*. 2021 emend.

The description of the genus *Mycobacterium* is as given by Meehan et al. (12). It comprises all the species included in the genera *Mycobacterium*, *Mycolicibacillus*, *Mycolicibacter,* and *Mycolicibacterium* described by Gupta et al. (17). The type species of the genus is *Mycobacterium tuberculosis* (Zopf 1883) Lehmann and Neumann 1896 [Approved Lists 1980 (55)].

### *Corynebacteriineae* corrig. Stackebrandt *et al*. 1997 emend. Zhi *et al*. 2009 emend.

The suborder *Corynebacteriineae* Stackebrandt et al. 1997 was proposed to contain the families *Corynebacteriaceae*, *Dietziaceae*, *Gordoniaceae*, *Mycobacteriaceae*, *Nocardiaceae,* and *Tsukamurellaceae* (54). Zhi et al. (61) emended the suborder by removing the family *Gordoniaceae* and adding the family *Segniliparaceae*. Goodfellow and Jones (62) elevated the suborder to the order *Corynebacteriales* including the families *Dietziaceae*, *Gordoniaceae*, *Mycobacteriaceae*, *Nocardiaceae*, *Segniliparaceae,* and *Tsukamurellaceae*. Later, the name of the order was changed to *Mycobacteriales* (63) to reflect the priority of *Mycobacteriales* Janke 1924 over *Corynebacteriales* Goodfellow and Jones 2012. The suborder *Corynebacteriineae* contains the families *Corynebacteriaceae*, *Dietziaceae,* and *Lawsonellaceae*. The estimated average genome size of the suborder is 2.5 and G + C content is 62. ± 6.2. The type genus is *Corynebacterium* Lehmann and Neumann 1896 [Approved Lists 1980 (55)].

## MATERIALS AND METHODS

### Genomes

A total of 272 genomes, each from a different *Mycobacteriales* species, were used in this study (Tables S1 and S2). Representatives of each genus and its internal diversity were selected according to the information in NCBI’s Taxonomy Browser (https://www.ncbi.nlm.nih.gov/taxonomy), published phylogenies, and the List of Prokaryotic Names with Standing in Nomenclature (https://www.bacterio.net/). Only type strains were used, except for *Mycobacterium cannetti*, *R. cerastii*, some *Nocardia* spp. (unavailable), and the unclassified *Rhodococcus* spp. The genome assemblies were from NCBI’s RefSeq collection unless otherwise stated. Species whose genome assemblies were deemed as contaminated, partial, anomalous, or containing many frameshifts, were excluded.

### Core-genome phylogenetic analysis

Bacterial phylogenies were determined from conserved protein markers identified with Get_Homologues v5.34.0 software (64) using the COG clustering algorithm (65) and BLASTp cutoffs of 1e-05 E-value and 75% coverage. The set of core-genome orthologs was aligned, filtered for recombinant or low-quality alignments rendering anomalous or poorly resolved trees, and combined in a supermatrix of top-scoring alignments using the Get_Phylomarkers pipeline (66). The supermatrix alignments for the *Mycobacteriales* (Fig. 1) and *Nocardiaceae* (Fig. S4) trees comprised 34 and 53 top markers, and 12,931 and 20,130 amino acid sites, respectively. ML phylogenies were inferred using the tree builder IQtree2 v2.0.5 (67) with LG + F + R9 best-fit substitution model selected by ModelFinder (68) and branch support determined by ultrafast bootstrap (69) (10,000 replicates) with option “–bnni“ on. Split phylogenetic networks were graphed using the SplitsTree5 v5.3.0 tool (70).

### Tree normalization and clustering

The branch length of the ML trees was normalized according to RED using the Phylorank software (https://github.com/donovan-h-parks/PhyloRank) (15). In the RED calculation, the root is set to 0 and each leaf to 1, with internal nodes being linearly interpolated between these values, allowing linear application of partitioning cutoffs regardless of potential differential rates of evolution between the lineages. The TreeCluster software was applied to the RED-normalized tree using the “Max Clade” clustering (35). This method splits the tree by uniformly applying a partitioning threshold (*t*) such that the maximum distance between two leaves in a cluster must be at most *t*. For each tree, a *t* range of 0.2 to 1 in increments of 0.01 was tested. For the purpose of genus demarcation, the selected *t* value aims at achieving optimal concordance with the established classical (pre-genomic) genera in the taxonomic context under study (see text above). Since the position of the genus rank in the tree hierarchy depends on the range of genetic distances of the diversity under analysis, the genus demarcation *t* value needs to be adjusted for each phylogeny.

### Genomic relatedness indices

The AAI of all conserved protein-coding sequences (CDS) shared between two genomes (6, 8, 36, 37) was calculated using “aai.rb” from the Kostas lab Enveomics Collection (71). The AF [average of the fractions between the length of all bidirectional best hits (BBH ≈ homologous genes) and the total length of all CDSs in reciprocal two-way comparisons] (13, 42) and gANI (sum of the nucleotide identities across all BBHs between the compared genomes divided by the sum of the lengths of the BBH genes) (13, 42) were determined using the ANI calculator tool (42). The gANI and AF pairwise comparisons are the average of the individual values resulting from using each of the two compared genomes as the first query. The POCP index was computed using Get_Homologues (command argument -P) (64). ML distance matrices were obtained as output files from IQtree (67).

### Network analysis

The taxonomic relationships of the analyzed bacteria were visualized in a three-dimensional context using network analysis of GRI and ML distance matrices using Biolayout software (http://biolayout.org/) (47). Upon .matrix file loading, the application of an increasing graph clustering threshold (ct) progressively disassembles the network graph into subnetworks of interconnected nodes (genomes) which can be related to different taxonomic ranks. At each ct cutoff, the subnetworks (taxonomic ranks) were validated using the Markov clustering algorithm (MCL) embedded in Biolayout, which non-subjectively divides the network graphs into discrete clusters of interrelated elements. Settings used: MCL algorithm inflation, 1.7; smallest cluster allowed, 2; rest of settings, default.

### Statistical analyses

The analysis and distributions of AAI values were performed using the stripchart function of the R package ggplot2. The statistical significance of the differences in the AAI values was determined in R by one-sample, one-tail Wilcoxon test against the value 65, or two-sample Wilcoxon test when comparing groups. The R version used was v4.3.0 (https://www.r-project.org).

## AFTER EPUB

*[This article was published on 5 October 2023 with typographical errors in the Taxonomic Descriptions. The errors were corrected in the current version, posted on 9 October 2023.]*

## SUPPLEMENTAL MATERIAL

Data Set S1 mbio.02207-23-s0001.xls ML distance and genomic relatedness index (GRI) matrices.

Supplemental material mbio.02207-23-s0002.pdf Supplemental text, figures, and tables.

Movie S1 mbio.02207-23-s0003.mp4 AAI network graph, genus level, *Mycobacteriales* lineage I

Movie S2 mbio.02207-23-s0004.mp4 AAI network graph, family level, *Mycobacteriales* lineage I.

Movie S3 mbio.02207-23-s0005.mp4 AAI network graph, suborder level, *Mycobacteriales* lineage I.

Movie S4 mbio.02207-23-s0006.mp4 AF network graph, suborder level, *Mycobacteriales* lineage I.

Movie S5 mbio.02207-23-s0007.mp4 ANI network graph, suborder level, *Mycobacteriales* lineage I.

Movie S6 mbio.02207-23-s0008.mp4 gANI network graph, suborder level, *Mycobacteriales* level I.

Movie S7 mbio.02207-23-s0009.mp4 ML distance network graph, suborder level, *Mycobacteriales* lineage I.

ASM does not own the copyrights to Supplemental Material that may be linked to, or accessed through, an article. The authors have granted ASM a non-exclusive, world-wide license to publish the Supplemental Material files. Please contact the corresponding author directly for reuse.
