# Supplementary material for: Mycobacteriales taxonomy using network analysis-aided, context-uniform phylogenomic approach for non-subjective genus demarcation
Source: mBio. 2023 Oct 5;14(5):e02207-23. doi: 10.1128/mbio.02207-23 (PMC10653829; doi:10.1128/mbio.02207-23)
Supplement: Supplemental material — Supplemental text, figures, and tables. [file mbio.02207-23-s0002.pdf]

## SUPPLEMENTAL MATERIAL FILES

- **Supplemental Material**: pdf file with Supplemental Text, Figures S1 to S6, and Tables S1 and S2.
- **Supplemental Dataset**: ML distance and genomic relatedness index (GRI) matrices used in network analyses in xls file.
- **Movie S1**: Genus-level AAI network graph (*Mycobacteriales* lineage I).
- **Movie S2**: Family-level AAI network graph (*Mycobacteriales* lineage I).
- **Movie S3**: Suborder-level AAI network graph (*Mycobacteriales* lineage I).
- **Movie S4**: AF network graph (suborder level, *Mycobacteriales* lineage I).
- **Movie S5**: ANI network graph (suborder level, *Mycobacteriales* lineage I).
- **Movie S6**: gANI network graph (suborder level, *Mycobacteriales* lineage I).
- **Movie S7**: ML distance network graph (suborder level, *Mycobacteriales* lineage I).

## SUPPLEMENTAL TEXT

***Nocardiaceae* phylogeny**. The *Nocardiacea*e ML tree (166 genomes) shows two main lines of descent: the genus *Nocardia*, and the rhodococci, comprised of the genus *Rhodococcus* "*sensu stricto*" and the "*fascians*" clade (genus *Rhodococcoides* gen. nov. proposed herein). There is also a minor grouping at the base of the *Nocardia* radiation comprising the monospecific genera *Skermania* and *Aldersonia,* and the three-species genus *Antrihabitans* (Fig. S4).

In the ML phylogenies, the rhodococci appear as an earlier evolving monophyletic grouping with greater internal diversity and more extended distances between its members compared to the nocardiae. This is also reflected in the network analyses, where the rhodococcal nodes are more loosely interconnected (Figs. 4, S6). In contrast, the *Nocardia* genus is consistently organized as a closely packed spherical cluster with equidistant nodes. This suggests a younger diversification, a more homogeneous ecological niche, less internal evolutionary bottlenecks, or a combination thereof.

Based on these differences, it could be justified classifying the rhodococcal clade as a separate family of the *Mycobacteriales* order. Each of the two main clades of this *Rhodococcaceae* family, i.e. the *Rhodococcus* and *Rhodococcoides* genera, is in turn subdivided into several monophyletic

subgeneric clades or sublineages, respectively five ("1" to "5") and two ("a" and "b") (Fig. S4). The more complex internal diversity of the *Rhodococcaceae* extends to basic attributes such as the genome size. Thus, in sublineage 4 (Fig. S4A) very large genomes of up to >8 Mbp (*Rhodococcus jostii*, *Rhodococcus opacus*, *Rhodococcus koreensis*, *Rhodococcus pseudokoreensis*, *Rhodococcus oxybenzonivorans*, *Rhodococcus wratislavensis*, two *Rhodococcus* spp.) coexist with significantly smaller genomes in the range of 4 to 5 Mbp (e.g. *Rhodococcus marinonascens*, *Rhodococcus rhodnii*, *Rhodococcus triatomae,* three *Rhodococcus* spp.) (see Fig. S2). Sublade 2 (*Prescottella*) also shows genome size heterogeneity, between 3.7-3.9 Mbp (*Rhodococcus xishaensis*, *Rhodococcus spongiicola*) and 5.4-5.7 (*R. agglutinans*, *Rhodococcus* sp. W8901) (see genome sizes in Table S2). These differences in DNA content are characteristic of the rhodococci and are mirrored by the unique metabolic versatility and niche adaptability of this bacterial group (1-7). The rhodococcal genome plasticity is critically underpinned by pools of circular and linear conjugative replicons of different sizes whose backbones have been actively exchanged and have acquired specific traits during niche-adaptive evolution (6, 8).

Visualization of the relationships of the minor taxons in the network analysis of the rhodococcal/nocardial raditation based on multiway ML distance- and GRI comparisons shows: (i) *Skermania piniformis* (*Nocardia pinensis*) DSM 43998$^{T}$ is most closely connected with *Nocardia* spp.; (ii) *Aldersonia* (*Rhodococcus*) *kunmingensis* DSM 45001$^{T}$ occupies an intermediate position between the *Nocardia* and *Rhodococcus* subnetworks (sometimes contributing with *Skermania* to link up the rhodococcal and nocardial clusters); (iii) *Antrihabitans* spp. are close to *Aldersonia*, with connecting edges with *Rhodococcus* nodes in the AAI, AF and gANI network graphs and *Nocardia* in the ML distance network graph (Fig. S6).

Two monospecies genera were excluded from the *Nocardiaceae* tree because they adversely affected its robustness (see Fig. S5): *Millisia brevis* NBRC 105863$^{T}$ and *Smaragdicoccus niigatensis* DSM 44881$^{T}$. In the network analyses, *M. brevis* was always associated with the rhododoccal

subnetwork, either equidistant to the *Rhodococcus* and "*fascians*"/*Rhodococcoides* clusters (gANI and AF networks), or connected with the latter (AAI). *S. niigatensis* always appears as a singleton, suggesting a more distant relationship.

In summary, our analyses suggest that the two major clades of the rhodococcal/nocardial radiation of the *Mycobacteriales* could be considered as separate families: a more diverse and longer-evolving *Rhodococcaceae* family, and a younger *Nocardiaceae* family comprised of the genus *Nocardia* (with two major sublineages) (Figs. 1, S4) and the monospecies genus *Skermania*. The exact affiliation of the *Aldersonia*, *Antrihabitans*, *Millisia* and *Smaragdicoccus* genera within the rhodococcal/nocardial radiation will need to await the genomic analysis of a larger diversity of isolates, not yet available.

## SUPPLEMENTAL FIGURES

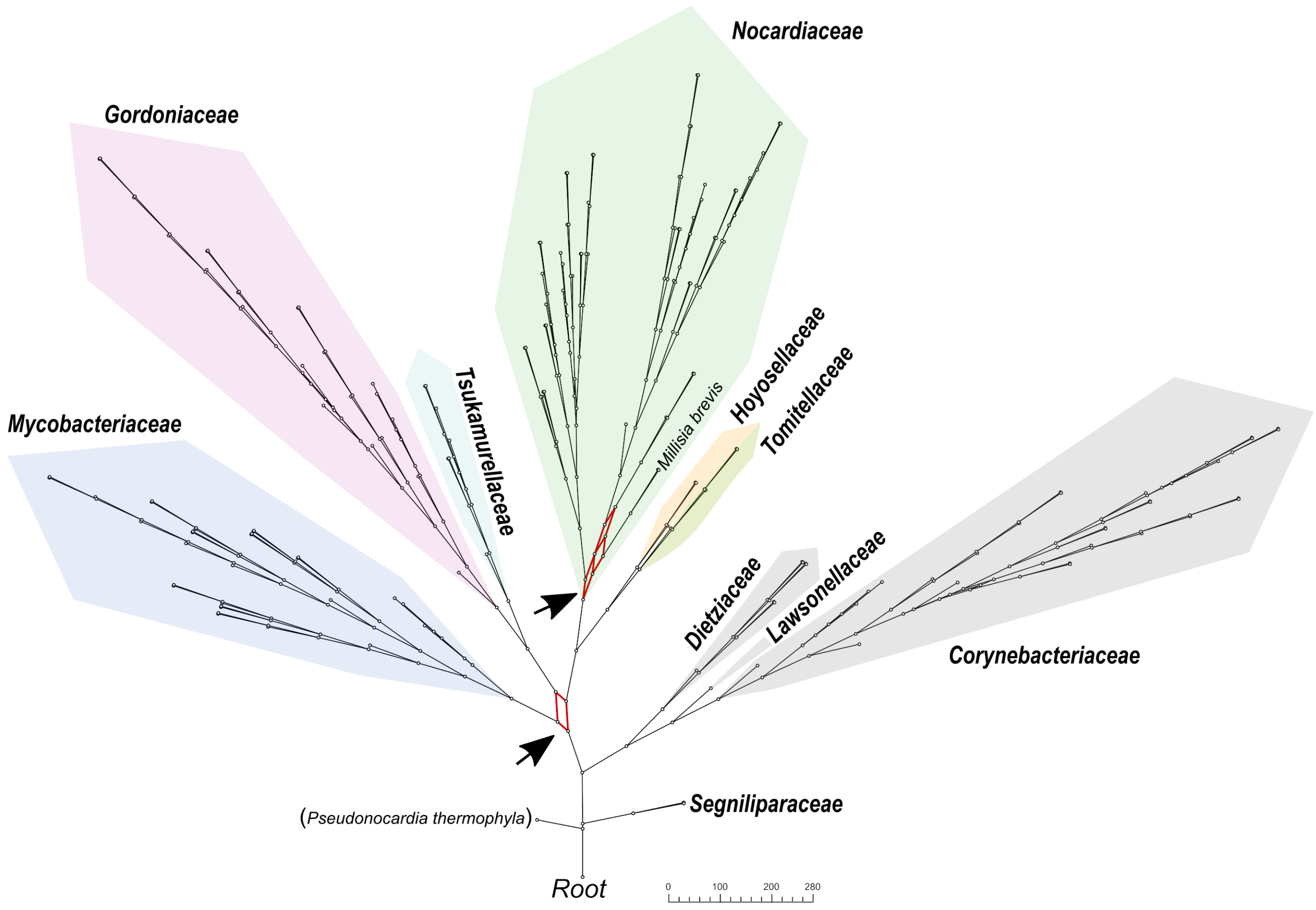


**FIG S1**. Split-network analysis of the *Mycobacteriales* ML tree in Fig. 1. Splits with a weight value lower than 20 were filtered. Alternative branchings are shown in red and indicated by black arrows. Most nodes in the ML tree are strongly supported with bootstrap values ≥80 (Fig. 1). The only exceptions are the relative instability of the *Gordoniaceae*/*Tsukarurellaceae* branching, which could stem from the base of either the *Nocardiaceae* or the *Mycobacteriaceae* (resulting in a node with bootstrap <70 in Fig. 1), and of the *Millisia brevis* branch at the base of the *Nocardiaceae*. See Fig. S5 for further details. Tree plotted using Splittree5 v5.3.0.

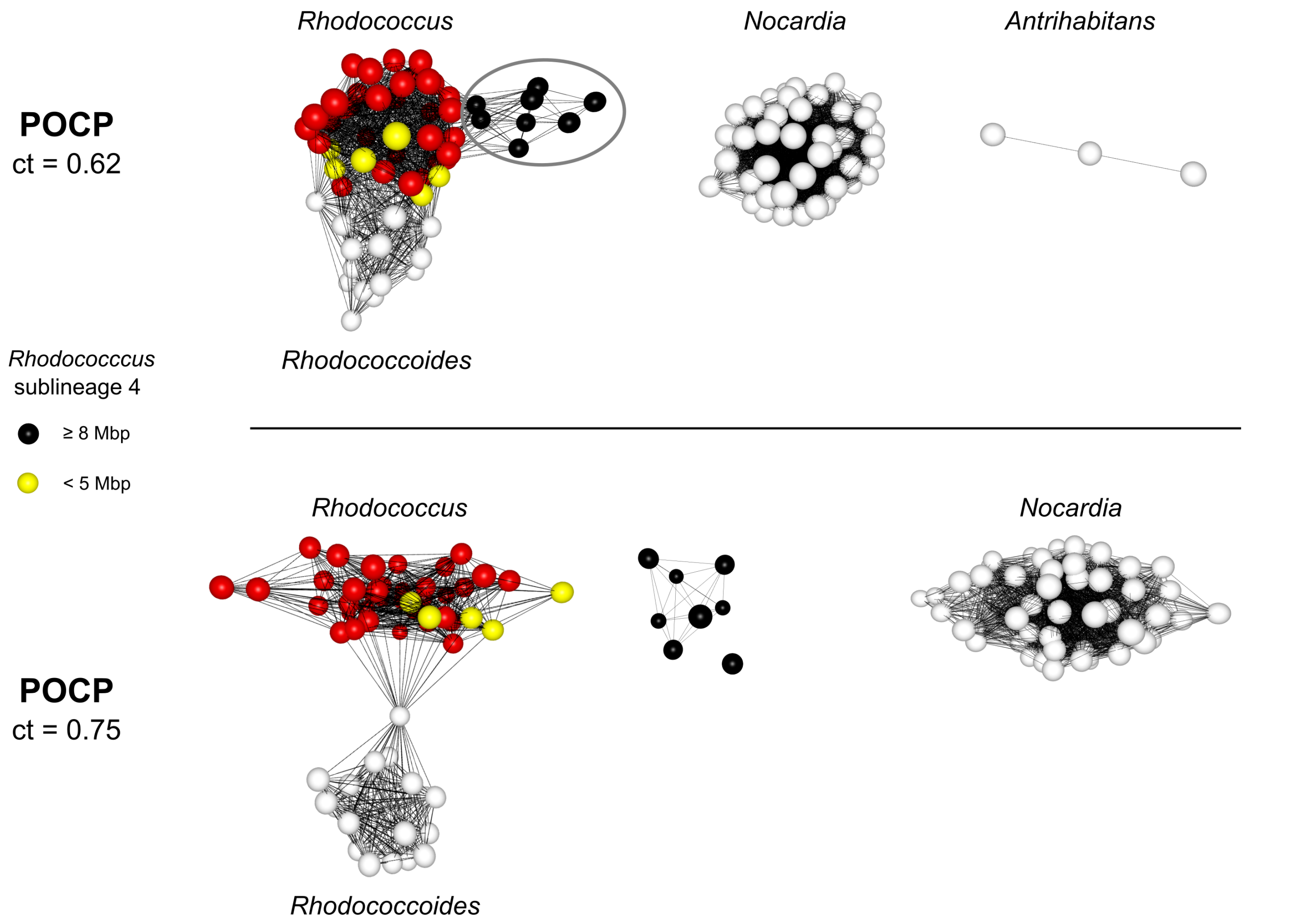


**FIG S2**. Network analysis of *Nocardiaceae* taxonomic relationships based on POCP genomic index. The figure illustrates that the POCP metric (the sum of BlastP orthologs identified in two-way genome comparisons divided by the total number of CDSs in the two genomes), as previously cautioned (9), could be potentially biased by differences in genome size. This is evident in the shown network graphs, where rhodococcal species with larger genomes (>8 Mbp) are clustered away from other members of their same sublineage (no. 4) despite all being phylogenetically closely related (see Figs. 1, 2). This observation underscores that genome size, although linked to the bacterial phylogeny at a broad scale (10), does not necessarily have taxonomic value at lower rank (e.g. genus) levels –reflecting that differential niche-adaptive genome expansion or contraction phenomena may occur in closely related bacteria (4, 11, 12). *Rhodococcus* (*sensu stricto*) nodes are represented in red, those belonging to sublineage no. 4 (see Figs. S4, S7) are in yellow when genomes are <5 Mbp (*Rhodococcus marinonascens*, *Rhodococcus rhodnii*, *Rhodococcus triatomae,* three *Rhodococcus* spp.), or black when genomes are ≥8 Mpb (*Rhodococcus jostii*, *Rhodococcus opacus*, *Rhodococcus koreensis*, *Rhodococcus pseudokoreensis*, *Rhodococcus oxybenzonivorans*, *Rhodococcus wratislavensis*, two *Rhodococcus* spp.). See also section "*Nocardiaceae* taxonomy" in Supplementary Text.

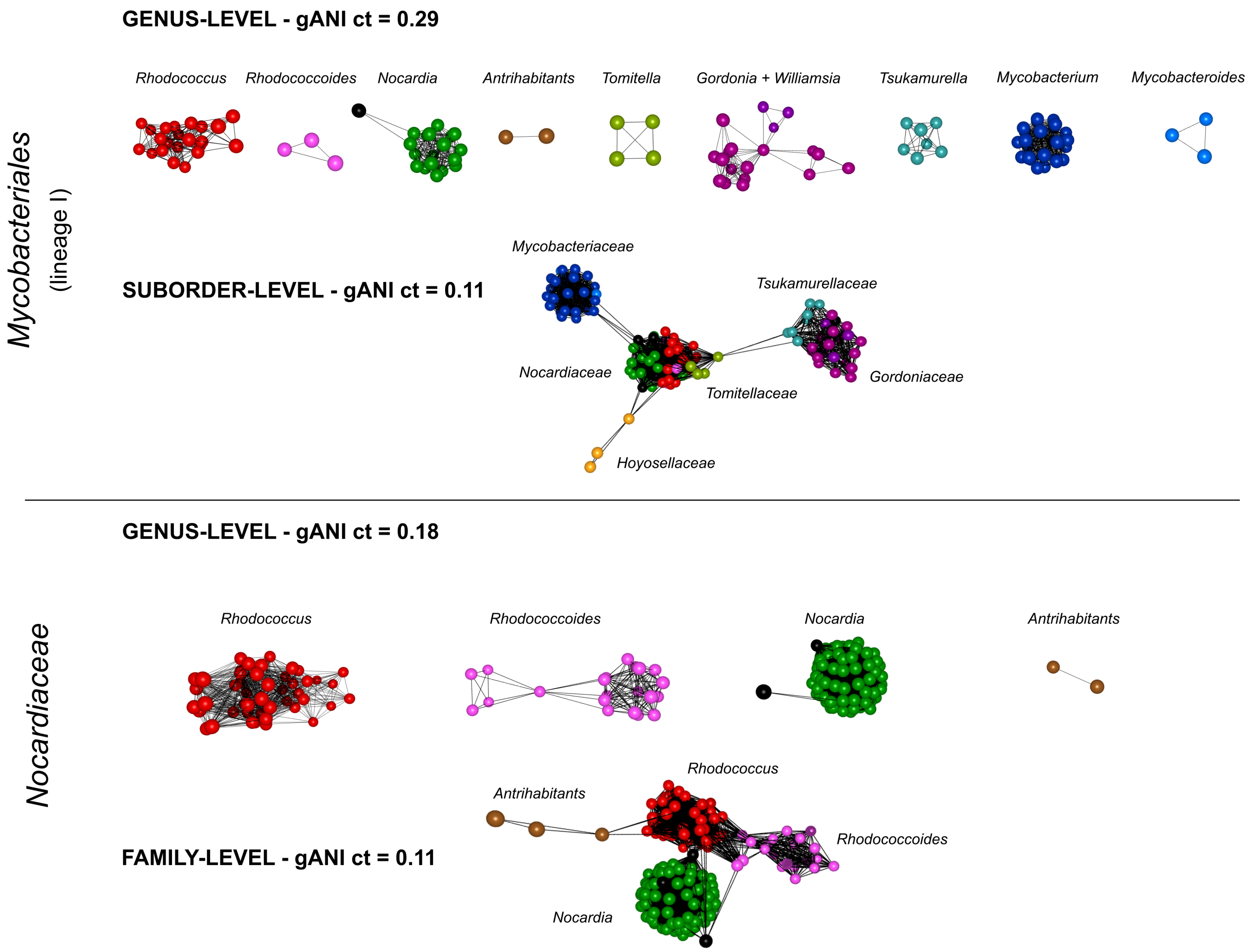


**FIG S3**. Taxonomic network analysis based on gANI genomic index at genus and supra-genus level. Top panel, *Mycobacteriales* lineage I; bottom panel, *Nocardiaceae*. At genus level, the gANI-based clustering gives subnetwork partitions fully consistent with those based on the AAI, AF and ML distance matrices (Figs. 3, 4), albeit with a smaller clustering threshold (ct) dynamic range, i.e. network over-fragmentation is rapidly reached as ct values increase. However, at low ct values, the gANI metric affords a good visualization of the taxonomic interconnections at supra-genus (i.e. suborder or family) level. See Movies S3 to S7 for 3D network animations showing the higher rank-level clustering resolution of AF, AAI, ML distance, gANI, and ANI.

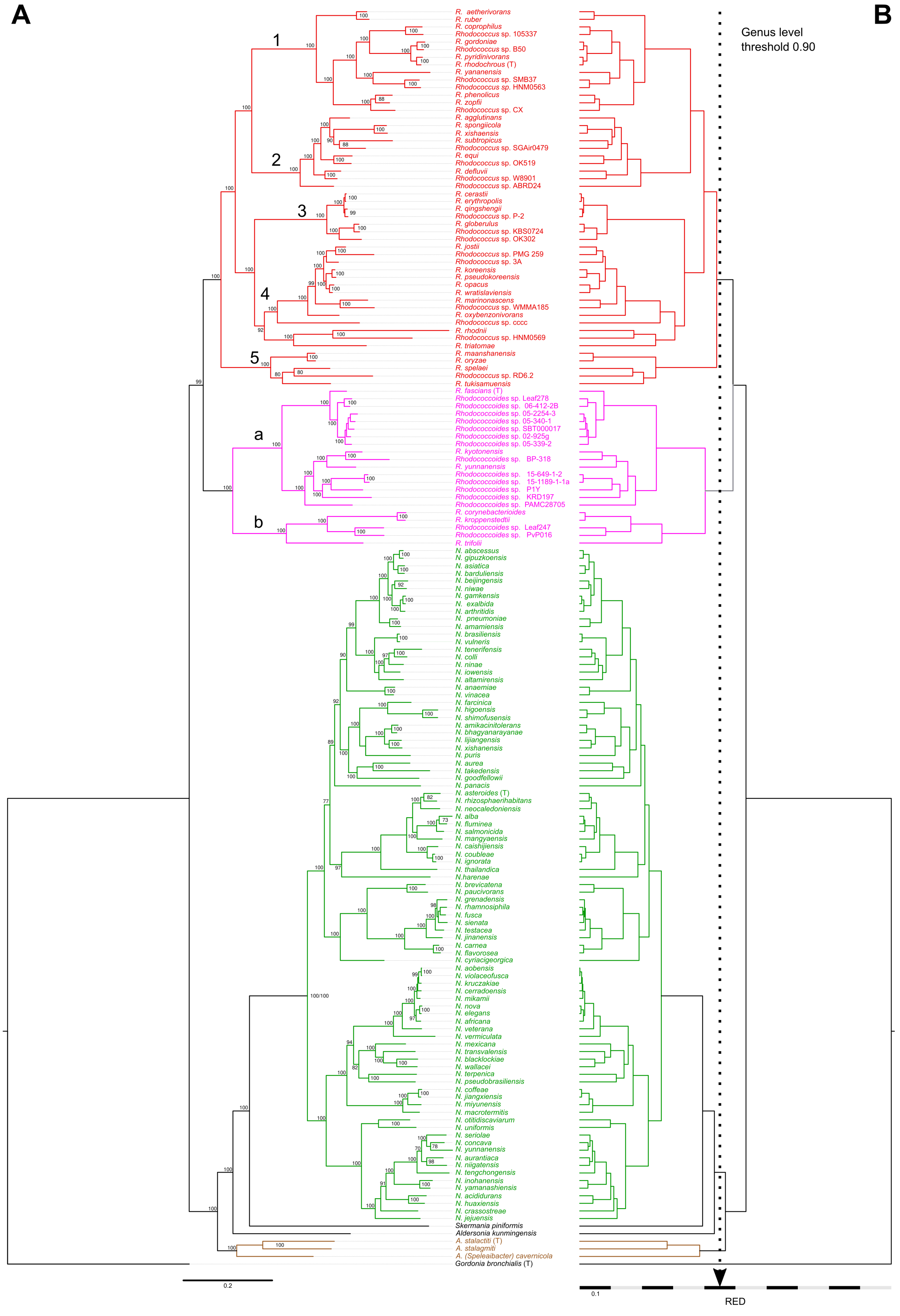
A
B
Genus level threshold 0.90
1
2
3
4
5
a
b
R. aetherivorans
R. ruber
R. coprophilus
Rhodococcus sp. 105337
R. gordoniae
Rhodococcus sp. B50
R. pyridinivorans
R. rhodochrous (T)
R. yananensis
Rhodococcus sp. SMB37
Rhodococcus sp. HNM0563
R. phenolicus
R. zopfii
Rhodococcus sp. CX
R. agglutinans
R. spongiicola
R. xishaensis
R. subtropicus
Rhodococcus sp. SGAir0479
R. equi
Rhodococcus sp. OK519
R. defluvii
Rhodococcus sp. W8901
Rhodococcus sp. ABRD24
R. cerastii
R. erythropolis
R. qingshengii
Rhodococcus sp. P-2
R. globerulus
Rhodococcus sp. KBS0724
Rhodococcus sp. OK302
R. jostii
Rhodococcus sp. PMG 259
Rhodococcus sp. 3A
R. koreensis
R. pseudokoreensis
R. opacus
R. wratislaviensis
R. marinonascens
Rhodococcus sp. WMMA185
R. oxybenzonivorans
Rhodococcus sp. cccc
R. rhodnii
Rhodococcus sp. HNM0569
R. triatomae
R. maanshanensis
R. oryzae
R. spelaei
Rhodococcus sp. RD6.2
R. tukisamuensis
R. fascians (T)
Rhodococcoides sp. Leaf278
Rhodococcoides sp. 06-412-2B
Rhodococcoides sp. 05-2254-3
Rhodococcoides sp. 05-340-1
Rhodococcoides sp. SBT000017
Rhodococcoides sp. 02-925g
Rhodococcoides sp. 05-339-2
R. kyotonensis
Rhodococcoides sp. BP-318
R. yunnanensis
Rhodococcoides sp. 15-649-1-2
Rhodococcoides sp. 15-1189-1-1a
Rhodococcoides sp. P1Y
Rhodococcoides sp. KRD197
Rhodococcoides sp. PAMC28705
R. corynebacterioides
R. kroppenstedtii
Rhodococcoides sp. Leaf247
Rhodococcoides sp. PvP016
R. trifolii
N. abscessus
N. gipuzkoensis
N. asiatica
N. barduliensis
N. beijingensis
N. niwae
N. gamkensis
N. exalbida
N. arthritidis
N. pneumoniae
N. amamiensis
N. brasiliensis
N. vulneris
N. tenerifensis
N. colli
N. ninae
N. iowensis
N. altamirensis
N. anaemiae
N. vinacea
N. farcinica
N. higoensis
N. shimofusensis
N. amikacinitolerans
N. bhagyanarayanae
N. lijiangensis
N. xishanensis
N. puris
N. aurea
N. takedensis
N. goodfellowii
N. panacis
N. asteroides (T)
N. rhizosphaerihabitans
N. neocaledoniensis
N. alba
N. fluminea
N. salmonicida
N. mangyaensis
N. caishijiensis
N. coubleae
N. ignorata
N. thailandica
N.harenae
N. brevicatena
N. paucivorans
N. grenadensis
N. rhamnosiphila
N. fusca
N. sienata
N. testacea
N. jinanensis
N. carnea
N. flavorosea
N. cyriacigeorgica
N. aobensis
N. violaceofusca
N. kruczakiae
N. cerradoensis
N. mikamii
N. nova
N. elegans
N. africana
N. veterana
N. vermiculata
N. mexicana
N. transvalensis
N. blacklockiae
N. wallacei
N. terpenica
N. pseudobrasiliensis
N. coffeae
N. jiangxiensis
N. miyunensis
N. macrotermitis
N. otitidiscaviarum
N. uniformis
N. seriolae
N. concava
N. yunnanensis
N. aurantiaca
N. niigatensis
N. tengchongensis
N. inohanensis
N. yamanashiensis
N. acididurans
N. huaxiensis
N. crassostreae
N. jejuensis
Skermania piniformis
Aldersonia kunmingensis
A. stalactiti (T)
A. stalagmiti
A. (Speleaibacter) cavernicola
Gordonia bronchialis (T)
0.2
0.1
RED

**FIG S4 (previous page)**. *Nocardiaceae* ML phylogeny and tree partitioning. Genera are color coded according to Fig. 1. Type species of each genus are indicated. Trees drawn using FigTree v1.4.4 (http://tree.bio.ed.ac.uk/software/figtree/). (A) ML phylogenetic tree based on a concatenated alignment of 53 conserved proteins from 165 *Nocardiaceae* genomes plus *Gordonia bronchialis* DSM 43247$^T$ as outgroup (Table S2). Tree constructed as in Fig. 1. Bootstraps ≥70% are shown (10,000 replicates); see Fig. S5B for bootstrap representation as a consensus phylogenetic network. Numbers (1 to 5) and lower case letters (a and b) indicate, respectively, the main sublineages of the *Rhodococcus* (*sensu stricto*) genus (1 = "*rhodochrous*" clade, 2 = "*equi*"/*Prescottella* clade, 3 = "*erythropolis*" clade, 4 = "*jostii*" clade, 5 = "*maanshanensis*" clade) and *Rhodococcoides* genus (a = "*fascians*" subclade, b = "*corynebacteroides*" subclade). Scale bar indicates number of amino acid substitutions per site. *Millisia brevis* NBRC 105863$^T$ and *Smaragdicoccus niigatensis* DSM 44881$^T$ were excluded from the phylogenetic analysis because they significantly reduced the bootstrap support of key basal branches due to a long-branch attraction effect; see Fig. S5 for details. (B) TreeCluster (13) partitioning of RED-normalized ML tree (see Fig. 2 legend). Genus-level tree clustering cutoff indicated by dotted arrow. RED scale, relative evolutionary divergence. Possible new species of the genus are *Rhodococcoides* sp. Leaf278, *Rhodococcoides* sp. Leaf247, *Rhodococcoides* sp. 06-412-2B, *Rhodococcoides* sp. 05-2254-3, *Rhodococcoides* sp. 15-649-1-2, *Rhodococcoides* sp. 05-340-1, *Rhodococcoides* sp. 05-339-2, *Rhodococcoides* sp. 02-925g, *Rhodococcoides* sp. 15-1189-1-1a, *Rhodococcoides* sp. P1Y, *Rhodococcoides* sp. SBT000017, *Rhodococcoides* sp. PAMC28705, *Rhodococcoides* sp. KRD197, *Rhodococcoides* sp. PvP016, and *Rhodococcoides* sp. BP-318 (all previously classified as *Rhodococcus* spp.). Type species: *Rhodococcoides fascians* comb. nov.

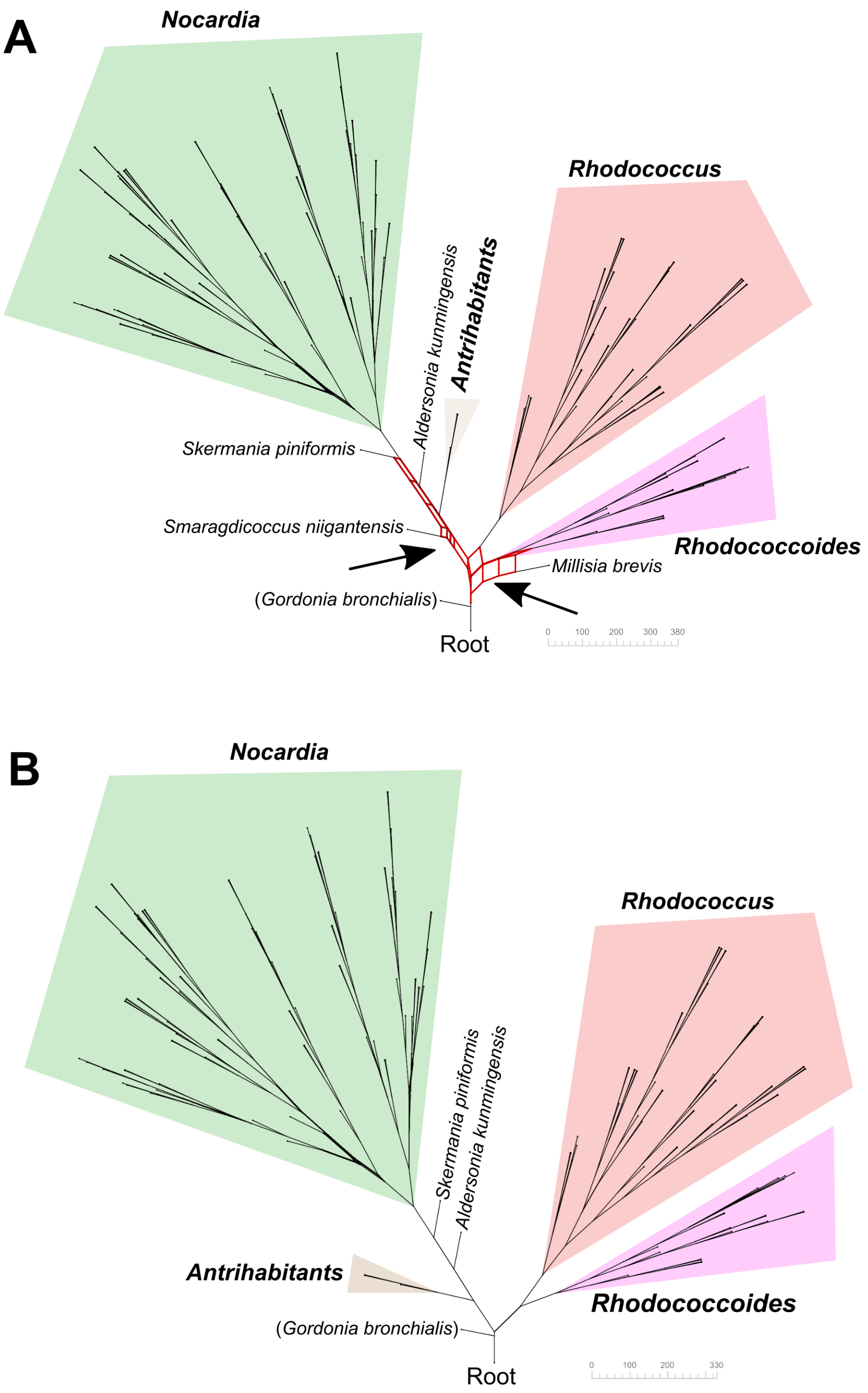


**FIG S5**. Split-network analysis of the *Nocardiaceae* ML tree in Fig. S4. Alternative branchings are shown in red and indicated by black arrows. Tree plotted using Splittree5 v5.3.0. (A) The presence of *Millisia brevis* NBRC 105863$^T$ and *Smaragdicoccus niigatensis* DSM 44881$^T$ in the phylogenetic analysis causes a generalized reduction in the bootstrap support of basal branches of the tree. The genomic indices of these two species indicate they are distantly related to the other *Nocardiaceae*, and both are located in relatively long branches in the ML tree (Fig. 1). This likely results in a Long Branch Attraction (LBA) effect due to random rather than phylogenetically informative substitutions, causing tree instability. (B) Same tree after removal of *M. brevis* NBRC 105863$^T$ and *S. niigatensis* DSM 44881$^T$ from the concatenated supermatrix.

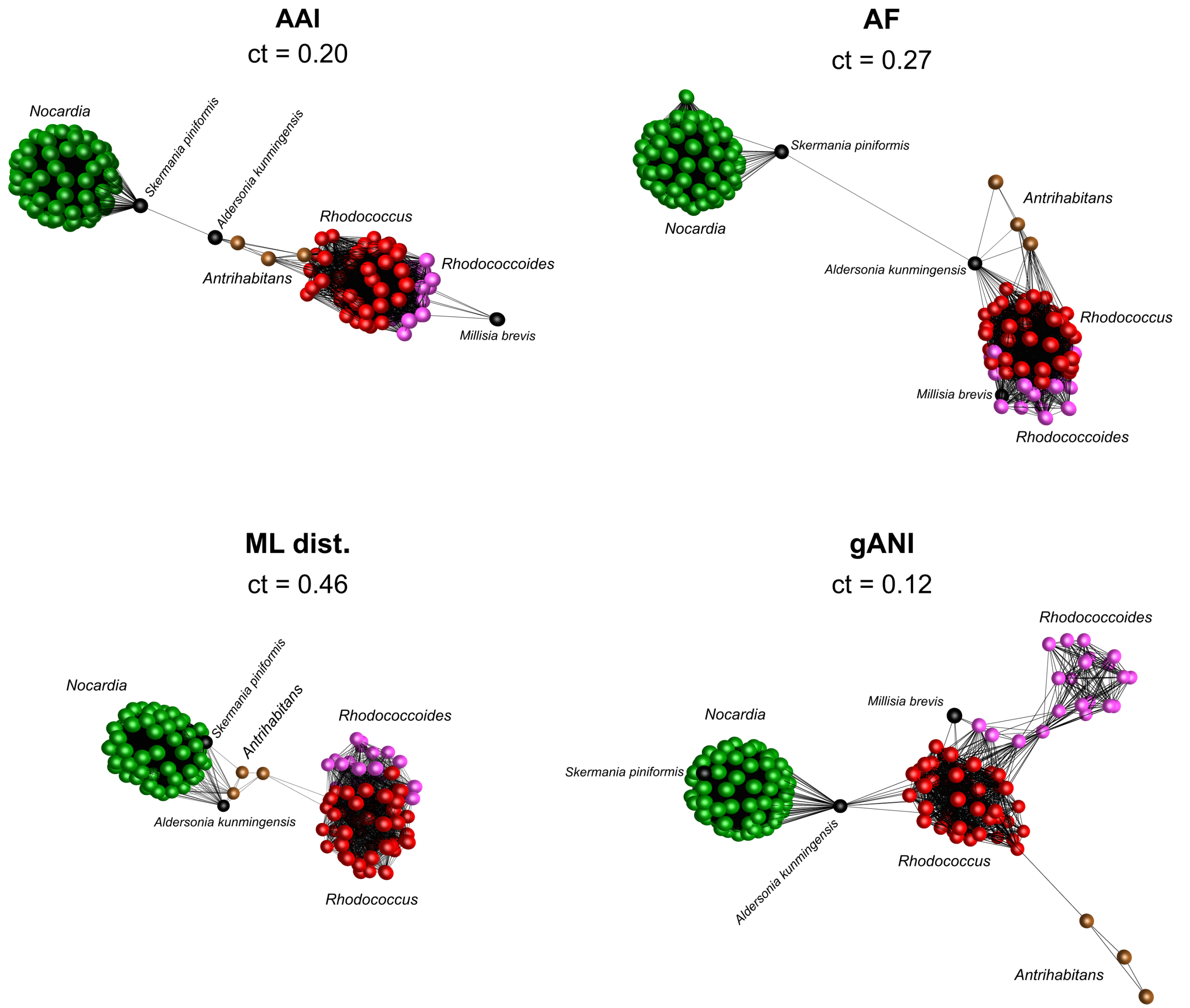


**FIG S6**. Taxonomic network analysis based on AAI, AF, gANI and ML distance indices showing the relationships of the monospecies genera *Aldersonia*, *Millisia* and *Skermania* with the rest of the *Nocardiaceae*. Clustering threshold (ct) applied at family level. Even at the lowest (less stringent) ct values, the more distant *Smaragdicoccus niigatensis* DSM 44881$^{T}$ appears as a singleton. With all metrics, raising the ct to genus level values isolates *Aldersonia*, *Millisia* and *Skermania* nodes in singletons.

**Supplementary Table S1.** Genomes used in *Mycobacteriales* phylogenomic analysis.

| Current name (as per NCBI) | Proposed name / emendation | Strain | Type | Accession | Genome size bp | GC % |
|---|---|---|---|---|---|---|
| *Aldersonia kunmingensis* | – | DSM 45001 | Yes | GCF_001646865.1 | 5,622,778 | 66 |
| *Antrihabitans stalactiti* | – | YC2-7 | Yes | GCF_012932915.1 | 6,760,149 | 64 |
| *Antrihabitans stalagmiti* | – | YC3-6 | Yes | GCF_016482825.1 | 6,393,340 | 63.5 |
| *Corynebacterium accolens* | – | ATCC 49725 | Yes | GCF_000159115.1 | 2,437,186 | 59.5 |
| *Corynebacterium afermentans* subsp. *lipophilum* | – | CCUG 32105 | Yes | GCF_015351335.1 | 2,332,830 | 65 |
| *Corynebacterium ammoniagenes* | – | DSM 20306 | Yes | GCF_001941425.1 | 2,790,185 | 55.5 |
| *Corynebacterium argentoratense* | – | DSM 44202 | Yes | GCF_000590555.1 | 2,031,902 | 59 |
| *Corynebacterium aurimucosum* | – | DSM 44532 | Yes | GCF_024138775.1 | 2,683,765 | 60.5 |
| *Corynebacterium bovis* | – | DSM 20582 | Yes | GCF_014191555.1 | 2,694,851 | 73 |
| *Corynebacterium casei* | – | LMG S-19264 | Yes | GCF_000550785.1 | 3,132,213 | 55.5 |
| *Corynebacterium coyleae* | – | DSM 44184 | Yes | GCF_900105505.1 | 2,568,936 | 61.5 |
| *Corynebacterium diphtheriae* | – | NCTC 11397 | Yes | GCF_001457455.1 | 2,463,666 | 53.5 |
| *Corynebacterium flavescens* | – | NBRC 14136 | Yes | GCF_006539465.1 | 2,643,964 | 60 |
| *Corynebacterium glutamicum* | – | ATCC 13032 | Yes | GCF_002847405.1 | 3,316,624 | 54 |
| *Corynebacterium humireducens* | – | NBRC 106098 | Yes | GCF_001571025.1 | 2,621,735 | 69 |
| *Corynebacterium imitans* | – | NCTC 13015 | Yes | GCF_900187215.1 | 2,565,606 | 64.5 |
| *Corynebacterium jeikeium* | – | ATCC 43734 | Yes | GCF_001999385.1 | 2,447,019 | 61.5 |
| *Corynebacterium macginleyi* | – | CCUG 32361 | Yes | GCF_003688935.1 | 2,419,073 | 57 |
| *Corynebacterium maris* | – | DSM 45190 | Yes | GCF_000442645.1 | 2,833,547 | 66.5 |
| *Corynebacterium otitidis* | – | ATCC 51513 | Yes | GCF_000296405.1 | 2,118,285 | 71.5 |
| *Corynebacterium phoceense* | – | MC1 | Yes | GCF_900092335.1 | 2,793,222 | 63 |
| *Corynebacterium pilosum* | – | NCTC 11862 | Yes | GCF_900447205.1 | 2,593,653 | 60.5 |
| *Corynebacterium propinquum* | – | DSM 44285 | Yes | GCF_000375525.1 | 2,458,635 | 56.5 |
| *Corynebacterium pseudodiphtheriticum* | – | DSM 44287 | Yes | GCF_000688415.1 | 2,261,008 | 55.5 |
| *Corynebacterium pseudotuberculosis* | – | DSM 20689 | Yes | GCF_003634885.1 | 2,338,546 | 52 |
| *Corynebacterium renale* | – | NCTC 7448 | Yes | GCF_900478035.1 | 2,335,089 | 59 |
| *Corynebacterium silvaticum* | – | KL0182 | Yes | GCF_004382825.2 | 2,554,413 | 54.5 |
| *Corynebacterium striatum* | – | ATCC 6940 | Yes | GCF_000159135.1 | 2,828,991 | 59.5 |
| *Corynebacterium ulcerans* | – | NCTC 7910 | Yes | GCF_900187135.1 | 2,453,761 | 53.5 |
| *Corynebacterium variabile* | – | NBRC 15286 | Yes | GCF_006539825.1 | 3,192,709 | 67.5 |
| *Corynebacterium xerosis* | – | NBRC 16721 | Yes | GCF_001552415.1 | 2,686,219 | 69.5 |
| *Dietzia alimentaria* | – | 72 | Yes | GCF_000226215.1 | 3,352,817 | 67.5 |
| *Dietzia cinnamea* | – | NBRC 102147 | Yes | GCF_001571065.1 | 3,598,827 | 71 |
| *Dietzia kunjamensis* | – | DSM 44907 | Yes | GCF_003610395.1 | 3,544,285 | 70.5 |

**Table S1** (cont.)

| Current name (as per NCBI) | Proposed name / emendation | Strain | Type | Accession | Genome size bp | GC % |
|---|---|---|---|---|---|---|
| *Dietzia maris* | – | IMV 195T | Yes | GCF_014144855.1 | 3,072,669 | 69.5 |
| *Dietzia massiliensis* | – | Marseille-Q0999 | Yes | GCF_018390595.1 | 3,579,718 | 70.5 |
| *Dietzia psychralcaliphila* | – | ILA-1 | Yes | GCF_003096095.1 | 3,904,836 | 69.5 |
| *Dietzia timorensis* | – | ID05-A0528 | Yes | GCF_001659785.1 | 3,607,892 | 65.5 |
| *Gordonia alkanivorans* | – | NBRC 16433 | Yes | GCF_000225505.1 | 5,071,550 | 67.5 |
| *Gordonia amarae* | – | NBRC 15530 | Yes | GCF_000241345.1 | 5,306,375 | 67.5 |
| *Gordonia amicalis* | – | DSM 44461 | Yes | GCF_012395955.1 | 4,925,831 | 67.5 |
| *Gordonia araii* | – | NBRC 100433 | Yes | GCF_000241265.1 | 3,906,554 | 68 |
| *Gordonia bronchialis* | – | DSM 43247 | Yes | GCF_000024785.1 | 5,290,012 | 67 |
| *Gordonia effusa* | – | NBRC 100432 | Yes | GCF_000241305.1 | 4,703,019 | 62.5 |
| *Gordonia hydrophobica* | – | NBRC 16057 | Yes | GCF_001592365.1 | 4,579,443 | 67.5 |
| *Gordonia malaquae* | – | NBRC 108250 | Yes | GCF_000344135.1 | 4,465,464 | 66.5 |
| *Gordonia neofelifaecis* | – | NRRL B-59395 | Yes | GCF_000192435.1 | 4,257,286 | 68 |
| *Gordonia otitidis* | – | NBRC 100426 | Yes | GCF_000248075.1 | 5,295,895 | 65.5 |
| *Gordonia paraffinivorans* | – | NBRC 108238 | Yes | GCF_000344155.1 | 4,632,332 | 69 |
| *Gordonia polyisoprenivorans* | – | ATCC BAA-14 | Yes | GCF_012396285.1 | 6,287,369 | 67 |
| *Gordonia rubripertincta* | – | ATCC 14352 | Yes | GCF_012396225.1 | 5,699,719 | 67.5 |
| *Gordonia sihwensis* | – | NBRC 108236 | Yes | GCF_000333035.1 | 4,140,890 | 68 |
| *Gordonia soli* | – | NBRC 108243 | Yes | GCF_000334455.1 | 5,375,815 | 67.5 |
| *Gordonia sputi* | – | ATCC 29627 | Yes | GCF_012396255.1 | 4,961,688 | 65.5 |
| *Gordonia terrae* | – | NCTC 10669 | Yes | GCF_901542405.1 | 5,706,840 | 68 |
| *Hoyosella altamirensis* | – | DSM 45258 | Yes | GCF_014191505.1 | 4,762,199 | 62 |
| *Hoyosella lacisalsi* | – | G463 | Yes | GCF_014841105.1 | 3,449,530 | 68.5 |
| *Hoyosella rhizosphaerae* | – | CGMCC 1.15478 | Yes | GCF_014643175.1 | 3,541,256 | 58.4 |
| *Jongsikchunia kroppenstedtii* | – | NP8-5 | Yes | GCF_000380485.1 | 4,225,368 | 67 |
| *Lawsonella clevelandensis* | – | X1036 | Yes | GCF_001293125.1 | 1,860,551 | 58.5 |
| *Millisia brevis* | – | NBRC 105863 | Yes | GCF_001552615.1 | 5,646,340 | 69 |
| *Mycobacterium avium* [a] | – | DSM 44156 | Yes | GCF_009741445.1 | 4,956,929 | 69.5 |
| *Mycobacterium canettii* [a] | – | CIPT 140010059 | No | GCF_000253375.1 | 4,482,059 | 65.5 |
| *Mycobacterium colombiense* [a] | – | CECT 3035 | Yes | GCF_000222105.3 | 5,579,559 | 68 |
| *Mycobacterium europaeum* [a] | – | DSM 45397 | Yes | GCF_002102155.1 | 5,630,674 | 68.5 |
| *Mycobacterium interjectum* [a] | – | ATCC 51457 | Yes | GCF_900078675.2 | 5,929,237 | 68 |
| *Mycobacterium intermedium* [a] | – | DSM 44049 | Yes | GCF_002086275.1 | 6,817,968 | 66 |
| *Mycobacterium intracellulare* [a] | – | ATCC 13950 | Yes | GCF_000277125.1 | 5,402,402 | 68 |
| *Mycobacterium lacus* [a] | – | JCM 15657 | Yes | GCF_010731535.1 | 5,092,988 | 67 |
| *Mycobacterium marinum* [a] | – | CCUG 20998 | Yes | GCF_003391395.1 | 6,453,310 | 65.5 |

**Table S1** (cont.)

| Current name (as per NCBI) | Proposed name / emendation | Strain | Type | Accession | Genome size bp | GC % |
|---|---|---|---|---|---|---|
| *Mycobacterium simiae* [a] | – | JCM 12377 | Yes | GCF_010727605.1 | 5,788,994 | 66 |
| *Mycobacterium tuberculosis* [a] | – | H37Rv | Yes | GCF_000195955.2 | 4,411,532 | 65.5 |
| *Mycobacterium xenopi* [a] | – | JCM 15661 | Yes | GCF_009936235.1 | 4,917,655 | 66 |
| *Mycobacteroides abscessus* [a] | *Mycobacteroides abscessus* [b] | ATCC 19977 | Yes | GCF_001942505.1 | 5,098,655 | 64 |
| *Mycobacteroides chelonae* [a] | *Mycobacteroides chelonae* [b] | CCUG 47445 | Yes | GCF_001632805.1 | 5,029,817 | 64 |
| *Mycobacteroides immunogenum* [a] | *Mycobacteroides immunogenum* [b] | CCUG 47286 | Yes | GCF_001605725.1 | 5,573,781 | 64.5 |
| *Mycolicibacillus parakoreensis* [a] | *Mycobacterium parakoreensis* [c] | DSM 45575 | Yes | GCF_022370835.1 | 3,936,223 | 70.5 |
| *Mycolicibacillus trivialis* [a] | *Mycobacterium trivialis* [c] | DSM 44153 | Yes | GCF_002102395.1 | 3,591,083 | 70.5 |
| *Mycolicibacter arupensis* [a] | *Mycobacterium arupensis* [c] | DSM 44942 | Yes | GCF_002086515.1 | 4,442,590 | 67.5 |
| *Mycolicibacter nonchromogenicus* [a] | *Mycobacterium nonchromogenicus* [c] | DSM 44164 | Yes | GCF_002101555.1 | 4,465,329 | 68 |
| *Mycolicibacter sinensis* [a] | *Mycobacterium sinensis* [c] | JDM601 | Yes | GCF_001552315.1 | 4,643,668 | 68.5 |
| *Mycolicibacter terrae* [a] | *Mycobacterium terrae* [c] | JCM 12143 | Yes | GCF_010726955.1 | 4,566,186 | 68.5 |
| *Mycolicibacterium canariasense* [a] | *Mycobacterium canariasense* [c] | CCUG 47953 | Yes | GCF_022179545.1 | 6,755,573 | 67.5 |
| *Mycolicibacterium chlorophenolicum* [a] | *Mycobacterium chlorophenolicum* [c] | NBRC 15527 | Yes | GCF_001050035.1 | 7,330,216 | 68.5 |
| *Mycolicibacterium fallax* [a] | *Mycobacterium fallax* [c] | JCM 6405 | Yes | GCF_000724065.1 | 4,156,821 | 70.5 |
| *Mycolicibacterium fortuitum* [a] | *Mycobacterium fortuitum* [c] | JCM 6387 | Yes | GCF_000805385.1 | 6,406,072 | 66 |
| *Mycolicibacterium komanii* [a] | *Mycobacterium komanii* [c] | GPK 1020 | Yes | GCF_001457595.1 | 5,378,970 | 67.5 |
| *Mycolicibacterium neoaurum* [a] | *Mycobacterium neoaurum* [c] | DSM 44074 | Yes | GCF_001552715.1 | 5,536,033 | 66.5 |
| *Mycolicibacterium setense* [a] | *Mycobacterium setense* [c] | DSM 45070 | Yes | GCF_002101775.1 | 6,265,049 | 66.5 |
| *Mycolicibacterium smegmatis* [a] | *Mycobacterium smegmatis* [c] | NCTC 8159 | Yes | GCF_000214155.1 | 6,983,267 | 67.5 |
| *Mycolicibacterium vaccae* [a] | *Mycobacterium vaccae* [c] | NBRC 14118 | Yes | GCF_010727125.1 | 6,168,821 | 68.5 |
| *Nocardia abscessus* | – | NBRC 100374 | Yes | GCF_000308455.1 | 8,410,401 | 68 |
| *Nocardia aobensis* | – | NBRC 100429 | Yes | GCF_000308375.1 | 7,548,062 | 68 |
| *Nocardia asteroides* | – | NCTC 11293 | Yes | GCF_900637185.1 | 6,983,363 | 70 |
| *Nocardia beijingensis* | – | NBRC 16342 | Yes | GCF_001612785.1 | 7,477,839 | 69 |
| *Nocardia brasiliensis* | – | NCTC 11294 | Yes | GCF_900454265.1 | 8,921,451 | 68 |
| *Nocardia brevicatena* | – | NBRC 12119 | Yes | GCF_000308495.1 | 7,007,170 | 67 |
| *Nocardia cyriacigeorgica* | – | DSM 44484 | Yes | GCF_005863225.1 | 6,262,819 | 68 |
| *Nocardia farcinica* | – | NCTC 11134 | Yes | GCF_001182745.1 | 6,459,780 | 70.5 |
| *Nocardia gamkensis* | – | DSM 44956 | Yes | GCF_012396055.1 | 7,728,041 | 68.5 |
| *Nocardia grenadensis* | – | NBRC 108939 | Yes | GCF_001613445.1 | 6,520,117 | 68 |
| *Nocardia harenae* | – | NBRC 108248 | Yes | GCF_001612885.1 | 6,144,291 | 72 |
| *Nocardia mexicana* | – | DSM 44952 | Yes | GCF_003350525.1 | 8,981,212 | 68.5 |
| *Nocardia nova* | – | NBRC 15556 | Yes | GCF_001613005.1 | 7,849,771 | 68 |
| *Nocardia otitidiscaviarum* | – | NCTC 1934 | Yes | GCF_900454305.1 | 7,536,042 | 69 |

**Table S1** (cont.)

| Current name (as per NCBI) | Proposed name / emendation | Strain | Type | Accession | Genome size bp | GC % |
|---|---|---|---|---|---|---|
| *Nocardia otitidiscaviarum* | – | NCTC 1934 | Yes | GCF_900454305.1 | 7,536,042 | 69 |
| *Nocardia pneumoniae* | – | NBRC 100136 | Yes | GCF_000308755.1 | 7,588,197 | 68 |
| *Nocardia seriolae* | – | NBRC 15557 | Yes | GCF_007990715.1 | 7,606,457 | 68.5 |
| *Nocardia tenerifensis* | – | DSM 44704 | Yes | GCF_003202065.1 | 9,840,405 | 68.5 |
| *Nocardia terpenica* | – | IFM 0706 | Yes | GCF_013186535.1 | 9,269,950 | 68.5 |
| *Nocardia testacea* | – | NBRC 100365 | Yes | GCF_000308775.1 | 7,268,410 | 68.5 |
| *Prescottella agglutinans* | *Rhodococcus agglutinans* [d] | CCTCC AB2014297 | Yes | GCF_004011865.1 | 5,425,108 | 69.2 |
| *Prescottella defluvii* | *Rhodococcus defluvii* [d] | Ca11 | Yes | GCF_000738775.1 | 4,404,788 | 69 |
| *Prescottella equi* | *Rhodococcus equi* [d] | DSM 20307 | Yes | GCF_002094305.1 | 6,433,900 | 70.5 |
| *Prescottella subtropicus* | *Rhodococcus subtropicus* [d] | C9-28 | Yes | GCF_005434945.1 | 5,303,098 | 70.5 |
| *Rhodococcus aetherivorans* | – | DSM 44752 | Yes | GCF_011058165.1 | 5,134,337 | 68.5 |
| *Rhodococcus coprophilus* | – | NBRC 100603 | Yes | GCF_001895045.1 | 5,199,710 | 69 |
| *Rhodococcus erythropolis* | – | NBRC 15567 | Yes | GCF_001552595.1 | 4,549,097 | 67 |
| *Rhodococcus fascians* | *Rhodococcoides fascians* [d] | NBRC 12155 | Yes | GCF_001894785.1 | 6,588,929 | 62.5 |
| *Rhodococcus globerulus* | – | NBRC 14531 | Yes | GCF_001894805.1 | 5,773,359 | 64.5 |
| *Rhodococcus gordoniae* | – | NCTC 13296 | Yes | GCF_900455725.1 | 6,735,237 | 67.9 |
| *Rhodococcus jostii* | – | DSM 44719 | Yes | GCF_900105375.1 | 4,871,915 | 68 |
| *Rhodococcus koreensis* | – | DSM 44498 | Yes | GCF_900105905.1 | 9,912,981 | 67 |
| *Rhodococcus kroppenstedtii* | *Rhodococcoides kroppenstedtii* [d] | DSM 44908 | Yes | GCF_900111805.1 | 10,311,574 | 67.5 |
| *Rhodococcus kyotonensis* | *Rhodococcoides kyotonensis* [d] | JCM 23211 | Yes | GCF_900188125.1 | 4,082,826 | 70 |
| *Rhodococcus maanshanensis* | – | NBRC 100610 | Yes | GCF_001894865.1 | 6,311,006 | 64 |
| *Rhodococcus marinonascens* | – | NBRC 14363 | Yes | GCF_001894885.1 | 5,673,710 | 69 |
| *Rhodococcus opacus* | – | DSM 43205 | Yes | GCF_001646735.1 | 4,924,407 | 64.5 |
| *Rhodococcus pyridinivorans* | – | DSM 44555 | Yes | GCF_900105195.1 | 8,534,314 | 67.5 |
| *Rhodococcus qingshengii* | – | JCM 15477 | Yes | GCF_001646745.1 | 5,262,484 | 68 |
| *Rhodococcus rhodnii* | – | ATCC 35071 | Yes | GCF_008011915.1 | 7,261,179 | 62.5 |
| *Rhodococcus rhodochrous* | – | NBRC 16069 | Yes | GCF_001047055.1 | 4,491,802 | 69.5 |
| *Rhodococcus ruber* | – | DSM 43338 | Yes | GCF_001646835.1 | 5,201,718 | 68 |
| *Rhodococcus triatomae* | – | DSM 44892 | Yes | GCF_014217785.1 | 4,772,018 | 68.5 |
| *Rhodococcus wratislaviensis* | – | NCTC 13229 | Yes | GCF_900455735.1 | 7,777,377 | 67.5 |
| *Rhodococcus yunnanensis* | *Rhodococcoides yunnanensis* [d] | NBRC 103083 | Yes | GCF_001895005.1 | 6,373,933 | 64 |
| *Rhodococcus zopfii* | – | ATCC 51349 | Yes | GCF_022134735.1 | 6,274,579 | 68 |
| *Segniliparus rotundus* | – | DSM 44985 | Yes | GCF_000092825.1 | 3,157,527 | 67 |

**Table S1** (cont.)

| Current name (as per NCBI)[a] | Proposed name / emendation | Strain | Type | Accession | Genome size bp | GC % |
|---|---|---|---|---|---|---|
| *Segniliparus rugosus* | – | ATCC BAA-974 | Yes | GCF_000185725.2 | 3,587,629 | 68 |
| *Skermania piniformis* | – | DSM 43998 | Yes | GCF_019285775.1 | 4,230,116 | 68.5 |
| *Smaragdicoccus niigatensis* | – | DSM 44881 | Yes | GCF_000380645.1 | 5,320,466 | 64.5 |
| *Tomitella biformata* | – | AHU 1821 | Yes | GCF_000524475.1 | 4,709,534 | 68 |
| *Tomitella cavernae* | – | JCM 18542 | Yes | GCF_016599145.1 | 4,021,385 | 70.5 |
| *Tomitella fengzijianii* | – | HY188 | Yes | GCF_007559025.1 | 4,013,250 | 71 |
| *Tomitella gaofuii* | – | HY172 | Yes | GCF_014126825.1 | 4,353,844 | 71 |
| *Tsukamurella conjunctivitidis* | – | HKU72 | Yes | GCF_007858475.1 | 5,076,401 | 71 |
| *Tsukamurella paurometabola* | – | DSM 20162 | Yes | GCF_000092225.1 | 4,479,724 | 68.5 |
| *Tsukamurella pseudospumae* | – | JCM 13375 | Yes | GCF_001575205.1 | 4,939,052 | 70 |
| *Tsukamurella pulmonis* | – | NCTC 13230 | Yes | GCF_900460155.1 | 4,773,464 | 71 |
| *Tsukamurella spumae* | – | DSM 44113 | Yes | GCF_012396015.1 | 4,461,018 | 70 |
| *Tsukamurella sputi* | – | HKU70 | Yes | GCF_007858445.1 | 4,810,120 | 70 |
| *Tsukamurella tyrosinosolvens* | – | CCUG 38499 | Yes | GCF_001575155.1 | 5,223,451 | 71 |
| *Williamsia deligens* | – | DSM 44902 | Yes | GCF_024171765.1 | 4,410,574 | 70 |
| *Williamsia herbipolensis* | – | ARP1 | Yes | GCF_000964005.1 | 4,744,957 | 68.5 |
| *Williamsia maris* | – | DSM 44693 | Yes | GCF_024171815.1 | 4,931,535 | 68 |
| *Williamsia muralis* | – | DSM 44343 | Yes | GCF_003634525.1 | 5,401,534 | 65 |

[a] Mycobacterial names remain listed in NCBI databases as per the five-genus scheme of Gupta et al. (ref. 14) i.e. *Mycobacterium*, *Mycobacteroides*, *Mycolicibacter, Mycolicibacillus*, *Mycolicibacterium*, although the *Mycobacteroides*, *Mycolicibacter, Mycolicibacillus*, and *Mycolicibacterium* circumscriptions were reclassified in 2021 back into *Mycobacterium*, and corresponding nomenclature emended, by Meehan et al. (ref. 15).

[b] Emendation to Meehan et al. nomenclature (ref. 15) whereby the genus *Mycobacteroides* proposed by Gupta et al. (ref. 14) is maintained for the basal mycobacterial sublineage containing *Mycobacterium abscessus*.

[c] Emended name as per Meehan et al. (ref. 15), confirmed by our study (except for the *Mycobacteroides* clade, which should have genus status).

[d] Revised rhodococcal nomenclature based on the findings in this study.

**Supplementary Table S2.** Genomes used in *Nocardiaceae* phylogenomic analysis.

| Current name | Proposed new name / emendation | Strain | Type | Accession | Genome size bp | GC % |
|---|---|---|---|---|---|---|
| *Aldersonia kunmingensis* | – | DSM 45001 | Yes | GCF_001646865.1 | 5,622,778 | 66 |
| *Antrihabitans stalactiti* | – | YC2-7 | Yes | GCF_012932915.1 | 6,760,149 | 64 |
| *Antrihabitans stalagmiti* | – | YC3-6 | Yes | GCF_016482825.1 | 6,393,340 | 63.5 |
| *Millisia brevis* | – | NBRC 105863 | Yes | GCF_001552615.1 | 5,646,340 | 69 |
| *Nocardia abscessus* | – | NBRC 100374 | Yes | GCF_000308455.1 | 8,410,401 | 68 |
| *Nocardia acididurans* | – | LPG 2 | Yes | GCF_016741815.1 | 8,382,712 | 68 |
| *Nocardia africana* | – | NBRC 100379 | Yes | GCF_001612635.1 | 7,810,982 | 68 |
| *Nocardia alba* | – | DSM 44684 | Yes | GCF_004339125.1 | 7,289,173 | 67.5 |
| *Nocardia altamirensis* | – | NBRC 108246 | Yes | GCF_001612685.1 | 9,829,580 | 67 |
| *Nocardia amamiensis* | – | NBRC 102102 | Yes | GCF_001612745.1 | 8,243,020 | 67.5 |
| *Nocardia amikacinitolerans* | – | DSM 45539 | Yes | GCF_024172045.1 | 7,663,905 | 68.5 |
| *Nocardia anaemiae* | – | NBRC 100462 | Yes | GCF_001612725.1 | 8,616,135 | 65.5 |
| *Nocardia aobensis* | – | NBRC 100429 | Yes | GCF_000308375.1 | 7,548,062 | 68 |
| *Nocardia arthritidis* | – | NBRC 100137 | Yes | GCF_001612765.1 | 7,124,189 | 68.5 |
| *Nocardia asiatica* | – | NBRC 100129 | Yes | GCF_000308415.1 | 8,461,316 | 68.5 |
| *Nocardia asteroides* | – | NCTC 11293 | Yes | GCF_900637185.1 | 6,983,363 | 70 |
| *Nocardia aurantiaca* | – | CT2-14 | Yes | GCF_009708175.1 | 7,376,943 | 68 |
| *Nocardia aurea* | – | SYSU K10002 | Yes | GCF_003123685.1 | 9,368,495 | 67.5 |
| *Nocardia barduliensis* | – | 335427 | Yes | GCF_013315795.1 | 8,495,341 | 68.5 |
| *Nocardia beijingensis* | – | NBRC 16342 | Yes | GCF_001612785.1 | 7,477,839 | 69 |
| *Nocardia bhagyanarayanae* | – | DSM 103495 | Yes | GCF_006716565.1 | 7,555,939 | 69 |
| *Nocardia blacklockiae* | – | N-17 | No | GCF_015477355.1 | 7,934,999 | 70 |
| *Nocardia brasiliensis* | – | NCTC11294 | Yes | GCF_900454265.1 | 8,921,451 | 68 |
| *Nocardia brevicatena* | – | NBRC 12119 | Yes | GCF_000308495.1 | 7,007,170 | 67 |
| *Nocardia caishijiensis* | – | DSM 44831 | Yes | GCF_009858255.1 | 6,310,860 | 68 |
| *Nocardia carnea* | – | NBRC 14403 | Yes | GCF_000308515.1 | 7,492,940 | 67 |
| *Nocardia cerradoensis* | – | NBRC 101014 | Yes | GCF_000308535.1 | 7,602,329 | 68 |
| *Nocardia coffeae* | – | CA2R105 | Yes | GCF_019890715.1 | 10,562,586 | 67 |
| *Nocardia colli* | – | CICC 11023 | Yes | GCF_008704205.1 | 10,032,764 | 67.5 |
| *Nocardia concava* | – | NBRC 100430 | Yes | GCF_000308815.1 | 8,933,351 | 67.5 |
| *Nocardia coubleae* | – | DSM 44960 | Yes | GCF_012395945.1 | 6,623,463 | 68 |
| *Nocardia crassostreae* | – | NBRC 100342 | Yes | GCF_001613405.1 | 8,293,148 | 67.5 |
| *Nocardia cyriacigeorgica* | – | DSM 44484 | Yes | GCF_005863225.1 | 6,262,819 | 68 |
| *Nocardia elegans* | – | NBRC 108235 | Yes | GCF_001612845.1 | 7,539,150 | 68 |
| *Nocardia exalbida* | – | NBRC 100660 | Yes | GCF_000308575.1 | 7,367,991 | 68.5 |
| *Nocardia farcinica* | – | NCTC 11134 | Yes | GCF_001182745.1 | 6,459,780 | 70.5 |
| *Nocardia flavorosea* | – | NBRC 108225 | Yes | GCF_001613385.1 | 7,440,171 | 67 |

**Table S2** (cont.)

| Current name | Proposed new name / emendation | Strain | Type | Accession | Genome size bp | GC % |
|---|---|---|---|---|---|---|
| *Nocardia fluminea* | – | DSM 44489 | Yes | GCF_002846365.1 | 8,057,701 | 67.5 |
| *Nocardia fusca* | – | NBRC 14340 | Yes | GCF_001618425.1 | 8,036,724 | 68 |
| *Nocardia gamkensis* | – | DSM 44956 | Yes | GCF_012396055.1 | 7,728,041 | 68.5 |
| *Nocardia gipuzkoensis* | – | 234509 | Yes | GCF_013839485.1 | 8,073,088 | 68.5 |
| *Nocardia goodfellowii* | – | DSM 45516 | Yes | GCF_017875645.1 | 7,982,434 | 67.5 |
| *Nocardia grenadensis* | – | NBRC 108939 | Yes | GCF_001613445.1 | 6,520,117 | 68 |
| *Nocardia harenae* | – | NBRC 108248 | Yes | GCF_001612885.1 | 6,144,291 | 72 |
| *Nocardia higoensis* | – | NBRC 100133 | Yes | GCF_000308595.1 | 6,980,705 | 69.5 |
| *Nocardia huaxiensis* | – | WCH-YHL-001 | Yes | GCF_013744875.1 | 8,339,910 | 68 |
| *Nocardia ignorata* | – | DSM 44496 | Yes | GCF_004362495.1 | 7,022,989 | 67.5 |
| *Nocardia inohanensis* | – | NBRC 100128 | Yes | GCF_001612945.1 | 8,118,011 | 68 |
| *Nocardia iowensis* | – | NRRL 5646 | Yes | GCF_019222765.1 | 8,948,074 | 67 |
| *Nocardia jejuensis* | – | NBRC 103114 | Yes | GCF_001613145.1 | 8,654,199 | 67.5 |
| *Nocardia jiangxiensis* | – | NBRC 101359 | Yes | GCF_000308615.1 | 10,445,918 | 67 |
| *Nocardia jinanensis* | – | CGMCC 4.3508 | Yes | GCF_014645735.1 | 8,000,126 | 67.5 |
| *Nocardia kruczakiae* | – | NBRC 101016 | Yes | GCF_001612965.1 | 7,324,382 | 68 |
| *Nocardia lijiangensis* | – | NBRC 108240 | Yes | GCF_001613045.1 | 8,159,797 | 68.5 |
| *Nocardia macrotermitis* | – | RB20 | Yes | GCF_009604405.1 | 8,539,590 | 67 |
| *Nocardia mangyaensis* | – | Y48 | Yes | GCF_001886715.1 | 7,310,115 | 68 |
| *Nocardia mexicana* | – | DSM 44952 | Yes | GCF_003350525.1 | 8,981,212 | 68.5 |
| *Nocardia mikamii* | – | NBRC 108933 | Yes | GCF_001613505.1 | 7,562,813 | 68 |
| *Nocardia miyunensis* | – | NBRC 108239 | Yes | GCF_001613065.1 | 10,520,083 | 67 |
| *Nocardia neocaledoniensis* | – | DSM 44717 | Yes | GCF_003182135.1 | 7,323,073 | 69.5 |
| *Nocardia niigatensis* | – | NBRC 100131 | Yes | GCF_000308655.1 | 8,224,214 | 68 |
| *Nocardia ninae* | – | NBRC 108245 | Yes | GCF_007990755.1 | 9,698,742 | 67 |
| *Nocardia niwae* | – | NBRC 108934 | Yes | GCF_001613465.1 | 7,305,361 | 69 |
| *Nocardia nova* | – | NBRC 15556 | Yes | GCF_001613005.1 | 7,849,771 | 68 |
| *Nocardia otitidiscaviarum* | – | NCTC 1934 | Yes | GCF_900454305.1 | 7,536,042 | 69 |
| *Nocardia panacis* | – | YIM PH 21724 | Yes | GCF_003598715.1 | 7,362,956 | 67 |
| *Nocardia paucivorans* | – | NBRC 100373 | Yes | GCF_000308675.1 | 6,001,070 | 66.5 |
| *Nocardia pneumoniae* | – | NBRC 100136 | Yes | GCF_000308755.1 | 7,588,197 | 68 |
| *Nocardia pseudobrasiliensis* | – | DSM 44290 | Yes | GCF_003350585.1 | 8,407,941 | 67.5 |
| *Nocardia puris* | – | DSM 44599 | Yes | GCF_003315035.1 | 7,694,151 | 70 |
| *Nocardia rhamnosiphila* | – | NBRC 108938 | Yes | GCF_001613485.1 | 7,749,274 | 68.5 |
| *Nocardia rhizosphaerihabitans* | – | CGMCC 4.7329 | Yes | GCF_014646295.1 | 7,679,796 | 68.5 |
| *Nocardia salmonicida* | – | NBRC 13393 | Yes | GCF_001613085.1 | 8,253,158 | 67 |
| *Nocardia seriolae* | – | NBRC 15557 | Yes | GCF_007990715.1 | 7,606,457 | 68.5 |

**Table S2** (cont.)

| Current name | Proposed new name / emendation | Strain | Type | Accession | Genome size bp | GC % |
|---|---|---|---|---|---|---|
| *Nocardia shimofusensis* | – | NBRC 100134 | Yes | GCF_001613125.1 | 6,331,681 | 69 |
| *Nocardia sienata* | – | NBRC 100364 | Yes | GCF_001613205.1 | 6,836,334 | 68 |
| *Nocardia takedensis* | – | NBRC 100417 | Yes | GCF_000308695.1 | 8,033,873 | 69.5 |
| *Nocardia tenerifensis* | – | DSM 44704 | Yes | GCF_003202065.1 | 9,840,405 | 68.5 |
| *Nocardia tengchongensis* | – | CFH S0057 | Yes | GCF_018362975.1 | 8,006,012 | 68.5 |
| *Nocardia terpenica* | – | IFM 0706 | Yes | GCF_013186535.1 | 9,269,950 | 68.5 |
| *Nocardia testacea* | – | NBRC 100365 | Yes | GCF_000308775.1 | 7,268,410 | 68.5 |
| *Nocardia thailandica* | – | NBRC 100428 | Yes | GCF_000308795.1 | 6,821,826 | 71.5 |
| *Nocardia transvalensis* | – | NBRC 15921 | Yes | GCF_000308875.1 | 8,383,303 | 69 |
| *Nocardia uniformis* | – | NBRC 13702 | Yes | GCF_001613345.1 | 8,766,665 | 66 |
| *Nocardia vermiculata* | – | NBRC 100427 | Yes | GCF_001613265.1 | 6,687,976 | 67 |
| *Nocardia veterana* | – | DSM 44445 | Yes | GCF_012396305.1 | 6,800,911 | 68 |
| *Nocardia vinacea* | – | NBRC 16497 | Yes | GCF_000308835.1 | 10,155,283 | 65.5 |
| *Nocardia violaceofusca* | – | NBRC 14427 | No | GCF_001613525.1 | 7,505,823 | 68 |
| *Nocardia vulneris* | – | NBRC 108936 | Yes | GCF_001613425.1 | 9,377,610 | 68 |
| *Nocardia wallacei* | – | FMUON74 | No | GCF_014466955.1 | 7,892,862 | 69 |
| *Nocardia xishanensis* | – | NBRC 101358 | Yes | GCF_001613365.1 | 7,694,937 | 68.5 |
| *Nocardia yamanashiensis* | – | NBRC 100130 | Yes | GCF_001613325.1 | 9,095,921 | 68 |
| *Nocardia yunnanensis* | – | CFH S0054 | Yes | GCF_003626895.1 | 7,931,441 | 68.5 |
| *Prescottella agglutinans* | *Rhodococcus agglutinans* | CCTCC AB2014297 | Yes | GCF_004011865.1 | 5,425,108 | 69 |
| *Prescottella defluvii* | *Rhodococcus defluvii* | Ca11 | Yes | GCF_000738775.1 | 5,134,337 | 68.5 |
| *Prescottella equi* | *Rhodococcus equi* | DSM 20307 | Yes | GCF_002094305.1 | 5,199,710 | 69 |
| *Prescottella subtropicus* | *Rhodococcus subtropicus* | C9-28 | Yes | GCF_005434945.1 | 4,404,788 | 69 |
| *Rhodococcus aetherivorans* | – | DSM 44752 | Yes | GCF_011058165.1 | 6,433,900 | 70.5 |
| *Rhodococcus cerastii* | – | IEGM 1243 | No | GCA_021026135.1 | 7,008,365 | 62.5 |
| *Rhodococcus coprophilus* | – | NBRC 100603 | Yes | GCF_001895045.1 | 4,549,097 | 67 |
| *Rhodococcus corynebacterioides* | *Rhodococcoides corynebacterioides* | NBRC 14404 | Yes | GCF_001894765.1 | 3,977,374 | 70.5 |
| *Rhodococcus erythropolis* | – | NBRC 15567 | Yes | GCF_001552595.1 | 6,588,929 | 62.5 |
| *Rhodococcus fascians* | *Rhodococcoides fascians* | NBRC 12155 | Yes | GCF_001894785.1 | 5,773,359 | 64.5 |
| *Rhodococcus globerulus* | – | NBRC 14531 | Yes | GCF_001894805.1 | 6,735,237 | 61.7 |
| *Rhodococcus gordoniae* | – | NCTC13296 | Yes | GCF_900455725.1 | 4,871,915 | 68 |
| *Rhodococcus jostii* | – | DSM 44719 | Yes | GCF_900105375.1 | 9,912,981 | 67 |
| *Rhodococcus koreensis* | – | DSM 44498 | Yes | GCF_900105905.1 | 10,311,574 | 67.5 |
| *Rhodococcus kroppenstedtii* | *Rhodococcoides kroppenstedtii* | DSM 44908 | Yes | GCF_900111805.1 | 4,082,826 | 70 |
| *Rhodococcus kyotonensis* | *Rhodococcoides kyotonensis* | JCM 23211 | Yes | GCF_900188125.1 | 6,311,006 | 64 |
| *Rhodococcus maanshanensis* | – | NBRC 100610 | Yes | GCF_001894865.1 | 5,673,710 | 69 |
| *Rhodococcus marinonascens* | – | NBRC 14363 | Yes | GCF_001894885.1 | 4,924,407 | 64.5 |

**Table S2** (cont.)

| Current name | Proposed new name / emendation | Strain | Type | Accession | Genome size bp | GC % |
|---|---|---|---|---|---|---|
| *Rhodococcus opacus* | – | DSM 43205 | Yes | GCF_001646735.1 | 8,534,314 | 67.5 |
| *Rhodococcus oryzae* | – | NEAU-CX67 | Yes | GCF_005049235.1 | 5,365,841 | 69 |
| *Rhodococcus oxybenzonivorans* | – | S2-17 | Yes | GCF_003130705.1 | 8,014,224 | 65.5 |
| *Rhodococcus phenolicus* | – | DSM 44812 | Yes | GCF_001646785.1 | 6,283,907 | 68.5 |
| *Rhodococcus pseudokoreensis* | – | R79 | Yes | GCF_017068395.1 | 9,866,560 | 67.5 |
| *Rhodococcus pyridinivorans* | – | DSM 44555 | Yes | GCF_900105195.1 | 5,262,484 | 68 |
| *Rhodococcus qingshengii* | – | JCM 15477 | Yes | GCF_001646745.1 | 7,261,179 | 62.5 |
| *Rhodococcus rhodnii* | – | ATCC 35071 | Yes | GCF_008011915.1 | 4,491,802 | 69.5 |
| *Rhodococcus rhodochrous* | – | NBRC 16069 | Yes | GCF_001047055.1 | 5,201,718 | 68 |
| *Rhodococcus ruber* | – | DSM 43338 | Yes | GCF_001646835.1 | 5,303,098 | 70.5 |
| *Rhodococcus spelaei* | – | C9-5 | Yes | GCF_006704125.1 | 4,805,991 | 69 |
| *Rhodococcus spongiicola* | – | LHW50502 | Yes | GCF_004011835.1 | 3,967,637 | 66.5 |
| *Rhodococcus triatomae* | – | DSM 44892 | Yes | GCF_014217785.1 | 4,772,018 | 68.5 |
| *Rhodococcus trifolii* | *Rhodococcoides trifolii* | CCM 7905 | Yes | GCF_014635345.1 | 5,289,197 | 65.5 |
| *Rhodococcus tukisamuensis* | – | JCM 11308 | Yes | GCF_900101735.1 | 5,489,009 | 70 |
| *Rhodococcus wratislaviensis* | – | NCTC13229 | Yes | GCF_900455735.1 | 7,777,377 | 67.5 |
| *Rhodococcus xishaensis* | – | LHW51113 | Yes | GCF_004011825.1 | 3,709,357 | 66.5 |
| *Rhodococcus yananensis* | – | FBM22-1 | Yes | GCF_020515525.1 | 4,246,627 | 68.5 |
| *Rhodococcus yunnanensis* | *Rhodococcoides yunnanensis* | NBRC 103083 | Yes | GCF_001895005.1 | 6,373,933 | 64 |
| *Rhodococcus zopfii* | – | ATCC 51349 | Yes | GCF_022134735.1 | 6,274,579 | 68 |
| *Rhodococcus* sp. | – | PMG 259 | n/a | GCA_004211695.1 | 8,466,826 | 66.5 |
| *Rhodococcus* sp. | – | AS06rmzACSIP_394 | n/a | GCA_012514505.1 | 4,944,147 | 64.5 |
| *Rhodococcus* sp. | – | RD6.2 | n/a | GCA_001040705.1 | 5,572,456 | 68.5 |
| *Rhodococcus* sp. | *Rhodococcoides* sp. | Leaf278 | n/a | GCA_001426085.1 | 5,721,797 | 64 |
| *Rhodococcus* sp. | *Rhodococcoides* sp. | Leaf247 | n/a | GCA_001426185.1 | 4,533,613 | 67 |
| *Rhodococcus* sp. | – | WMMA185 | n/a | GCA_001767395.1 | 4,444,448 | 64 |
| *Rhodococcus* sp. | – | OK302 | n/a | GCA_002245895.1 | 6,790,176 | 61.5 |
| *Rhodococcus* sp. | *Rhodococcoides* sp. | 06-412-2B | n/a | GCA_002258385.1 | 6,359,028 | 64 |
| *Rhodococcus* sp. | *Rhodococcoides* sp. | 05-2254-3 | n/a | GCA_002258555.1 | 5,887,027 | 64.5 |
| *Rhodococcus* sp. | *Rhodococcoides* sp. | 15-649-1-2 | n/a | GCA_002258685.1 | 5,784,253 | 64.5 |
| *Rhodococcus* sp. | *Rhodococcoides* sp. | 05-340-1 | n/a | GCA_002258925.1 | 5,983,220 | 64.5 |
| *Rhodococcus* sp. | *Rhodococcoides* sp. | 05-339-2 | n/a | GCA_002258965.1 | 5,731,661 | 65 |
| *Rhodococcus* sp. | *Rhodococcoides* sp. | 02-925g | n/a | GCA_002259105.1 | 5,747,931 | 64.5 |
| *Rhodococcus* sp. | *Rhodococcoides* sp. | 15-1189-1-1a | n/a | GCA_002259155.1 | 6,236,672 | 64.5 |
| *Rhodococcus* sp. | – | OK519 | n/a | GCA_003051005.1 | 5,278,128 | 68 |
| *Rhodococcus* sp. | *Rhodococcoides* sp. | P1Y | n/a | GCA_003641205.1 | 5,868,661 | 63 |
| *Rhodococcus* sp. | *Rhodococcoides* sp. | SBT000017 | n/a | GCA_003688915.1 | 5,648,223 | 64.5 |

**Table S2** (cont.)

| Current name | Proposed new name / emendation | Strain | Type | Accession | Genome size bp | GC % |
|---|---|---|---|---|---|---|
| *Rhodococcus* sp. | – | ABRD24 | n/a | GCA_004328705.1 | 5,098,030 | 66.5 |
| *Rhodococcus* sp. | – | SMB37 | n/a | GCA_004345605.1 | 6,225,368 | 65.5 |
| *Rhodococcus* sp. | *Rhodococcoides* sp. | PAMC28705 | n/a | GCA_004795875.1 | 4,727,363 | 62 |
| *Rhodococcus* sp. | – | SGAir0479 | n/a | GCA_005484805.1 | 4,974,457 | 69.5 |
| *Rhodococcus* sp. | – | KBS0724 | n/a | GCA_005938745.2 | 7,254,422 | 61.5 |
| *Rhodococcus* sp. | – | HNM0563 | n/a | GCA_012726195.1 | 5,544,902 | 65.5 |
| *Rhodococcus* sp. | – | HNM0569 | n/a | GCA_012726205.1 | 4,408,839 | 69 |
| *Rhodococcus* sp. | – | 105337 | n/a | GCA_012844225.1 | 4,461,775 | 67.5 |
| *Rhodococcus* sp. | – | W8901 | n/a | GCA_013348805.1 | 5,713,496 | 68 |
| *Rhodococcus* sp. | – | B50 | n/a | GCA_013602415.2 | 6,157,152 | 67.5 |
| *Rhodococcus* sp. | – | 3A | n/a | GCA_014230115.1 | 8,742,699 | 67 |
| *Rhodococcus* sp. | – | CX | n/a | GCA_015865005.1 | 6,547,864 | 68.5 |
| *Rhodococcus* sp. | – | P-2 | n/a | GCA_016598535.1 | 6,340,135 | 62.5 |
| *Rhodococcus* sp. | *Rhodococcoides* sp. | KRD197 | n/a | GCA_017168235.1 | 5,564,651 | 64 |
| *Rhodococcus* sp. | *Rhodococcoides* sp. | PvP016 | n/a | GCA_017832185.1 | 4,502,611 | 68.5 |
| *Rhodococcus* sp. | *Rhodococcoides* sp. | BP-318 | n/a | GCA_019834925.1 | 5,289,915 | 65 |
| *Skermania piniformis* | – | DSM 43998 | Yes | GCF_019285775.1 | 4,230,116 | 68.5 |
| *Smaragdicoccus niigatensis* | – | DSM 44881 | Yes | GCF_000380645.1 | 5,320,466 | 64.5 |
| *Speleaibacter cavernicola* | *Antrihabitans cavernicola* | C1-24 | Yes | GCF_008297975.1 | 5,660,905 | 64.5 |

## SUPPLEMENTAL REFERENCES


1. van der Geize R, Dijkhuizen L. 2004. Harnessing the catabolic diversity of rhodococci for environmental and biotechnological applications. Curr Opin Microbiol 7: 255-61.
2. Larkin MJ, Kulakov LA, Allen CC. 2005. Biodegradation and *Rhodococcus* - masters of catabolic versatility. Curr Opin Biotechnol 16: 282-90.
3. McLeod MP, Warren RL, Hsiao WW, Araki N, Myhre M, Fernandes C, Miyazawa D, Wong W, Lillquist AL, Wang D, Dosanjh M, Hara H, Petrescu A, Morin RD, Yang G, Stott JM, Schein JE, Shin H, Smailus D, Siddiqui AS, Marra MA, Jones SJ, Holt R, Brinkman FS, Miyauchi K, Fukuda M, Davies JE, Mohn WW, Eltis LD. 2006. The complete genome of *Rhodococcus* sp. RHA1 provides insights into a catabolic powerhouse. Proc Natl Acad Sci U S A 103: 15582-7.
4. Letek M, Gonzalez P, Macarthur I, Rodriguez H, Freeman TC, Valero-Rello A, Blanco M, Buckley T, Cherevach I, Fahey R, Hapeshi A, Holdstock J, Leadon D, Navas J, Ocampo A, Quail MA, Sanders M, Scortti MM, Prescott JF, Fogarty U, Meijer WG, Parkhill J, Bentley SD, Vazquez-Boland JA. 2010. The genome of a pathogenic rhodococcus: cooptive virulence underpinned by key gene acquisitions. PLoS Genet 6: e1001145.
5. Kuyukina MS, Ivshina IB. 2019. Bioremediation of contaminated environments using *Rhodococcus*. Biology of *Rhodococcus*, pp. 231-270. *In* Alvarez HM (ed) Biology of *Rhodococcus*. Microbiology Monographs, Springer.
6. Vazquez-Boland JA, Meijer WG. 2019. The pathogenic actinobacterium *Rhodococcus equi*: what's in a name? Mol Microbiol 112: 1-15.
7. Hernández MA, Alvarez HM, Lanfranconi MP, Silva RA, Herrero OM, Villalba MS. 2019. Central metabolism of species of the genus *Rhodococcus*. pp. 61-85. *In* Alvarez HM (ed) Biology of *Rhodococcus*. Microbiology Monographs, Springer.
8. Larkin MJ, Kulakov LA, Allen CCR. 2010. Genomes and plasmids in *Rhodococcus*, p 73-90. *In* Alvarez HM (ed) Biology of *Rhodococcus*. Microbiology Monographs, Springer.
9. Qin Q-L, Xie B-B, Zhang X-Y, Chen X-L, Zhou B-C, Zhou J, Oren A, Zhang Y-Z. 2014. A proposed genus boundary for the prokaryotes based on genomic insights. J. Bacteriol 196: 2210-2215.
10. Martinez-Gutierrez CA, Aylward FO. 2022. Genome size distributions in bacteria and archaea are strongly linked to evolutionary history at broad phylogenetic scales. PLoS Genetics 18: e1010220.
11. Cole S, Eiglmeier K, Parkhill J, James K, Thomson N, Wheeler P, Honore N, Garnier T, Churcher C, Harris D. 2001. Massive gene decay in the leprosy bacillus. Nature 409: 1007-1011.
12. Stinear TP, Seemann T, Pidot S, Frigui W, Reysset G, Garnier T, Meurice G, Simon D, Bouchier C, Ma L. 2007. Reductive evolution and niche adaptation inferred from the genome of *Mycobacterium ulcerans*, the causative agent of Buruli ulcer. Genome Res 17: 192-200.
13. Balaban M, Moshiri N, Mai U, Jia X, Mirarab S. 2019. TreeCluster: Clustering biological sequences using phylogenetic trees. PloS One 14: e0221068.
14. Gupta RS, Lo B, Son J. 2018. Phylogenomics and comparative genomic studies robustly support division of the genus *Mycobacterium* into an emended genus *Mycobacterium* and four novel genera. Front Microbiol 9: 67.
15. Meehan CJ, Barco RA, Loh Y-HE, Cogneau S, Rigouts L. 2021. Reconstituting the genus *Mycobacterium*. Int J Syst Evol Microbiol 71: 004922.
